# Anti-Neoplastic Cytotoxicity of Gemcitabine-(C_4_-*amide*)-[anti-EGFR] in Dual-combination with Epirubicin-(C_3_-*amide*)-[anti-HER2/*neu*] against Chemotherapeutic-Resistant Mammary Adenocarcinoma (SKBr-3) and the Complementary Effect of Mebendazole

**DOI:** 10.17303/jcrto.2014.203

**Published:** 2014-04-09

**Authors:** CP Coyne, Toni Jones, Ryan Bear

**Affiliations:** Department of Basic Sciences, College of Veterinary Medicine at Wise Center, Mississippi State University, Mississippi State, Mississippi 39762, USA.

## Abstract

**Aims:**

Delineate the feasibility of simultaneous, dual selective “targeted” chemotherapeutic delivery and determine if this molecular strategy can promote higher levels anti-neoplastic cytotoxicity than if only one covalent immunochemotherapeutic is selectively “targeted” for delivery at a single membrane associated receptor over-expressed by chemotherapeutic-resistant mammary adenocarcinoma.

**Methodology:**

Gemcitabine and epirubicin were covalently bond to anti-EGFR and anti-HER2/*neu* utilizing a rapid multi-phase synthetic organic chemistry reaction scheme. Determination that 96% or greater gemcitabine or epirubicin content was covalently bond to immunoglobulin fractions following size separation by micro-scale column chromatography was established by methanol precipitation analysis. Residual binding-avidity of gemcitabine-(C_4_-*amide*)-[anti-EG-FR] applied in dual-combination with epirubicin-(C_3_-*amide*)-[anti-HER2/*neu*] was determined by cell-ELIZA utilizing chemotherapeutic-resistant mammary adenocarcinoma (SKBr-3) populations. Lack of fragmentation or polymerization was validated by SDS-PAGE/immunodetection/chemiluminescent autoradiography. Anti-neoplastic cytotoxic potency was determined by vitality stain analysis of chemotherapeutic-resistant mammary adenocarcinoma (SKBr-3) monolayers known to uniquely over-express EGFR (2 × 10^5^/cell) and HER2/*neu* (1 × 10^6^/cell) receptor complexes. The covalent immunochemotherapeutics gemcitabine-(C_4_-*amide*)-[anti-EGFR] and epirubicin-(C_3_-*amide*)-[anti-HER2/*neu*] were applied simultaneously in dual-combination to determine their capacity to collectively evoke elevated levels of anti-neoplastic cytotoxicity. Lastly, the tubulin/microtubule inhibitor mebendazole evaluated to determine if it’s potential to complemented the anti-neoplastic cytotoxic properties of gemcitabine-(C4-*amide*)-[anti-EGFR] in dual-combination with epirubicin-(C_3_-*amide*)-[*anti-HER2/neu*].

**Results:**

Dual-combination of gemcitabine-(C_4_-*amide*)-[anti-EGFR] with epirubicin-(C_3_-*amide*)-[anti-HER2/*neu*] produced greater levels of anti-neoplastic cytotoxicity than either of the covalent immunochemotherapeutics alone. The benzimidazole microtubule/tubulin inhibitor, mebendazole complemented the anti-neoplastic cytotoxicity of gemcitabine-(C_4_-*amide*)-[anti-EGFR] in dual-combination with epirubicin-(C_3_-*amide*)-[*anti-HER2/neu*].

**Conclusions:**

The dual-combination of gemcitabine-(C_4_-*amide*)-[anti-EGFR] with epirubicin-(C_3_-*amide*)-[anti-HER2/*neu*] produced higher levels of selectively “targeted” anti-neoplastic cytotoxicity against chemotherapeutic-resistant mammary adenocarcinoma (SKBr-3) than either covalent immunochemotherapeutic alone. The benzimidazole tubulin/microtubule inhibitor, mebendazole also possessed anti-neoplastic cytotoxicity against chemotherapeutic-resistant mammary adenocarcinoma (SKBr-3) and complemented the potency and efficacy of gemcitabine-(C_4_-*amide*)-[anti-EGFR] in dual-combination with epirubicin-(C_3_-*amide*)-[*anti-HER2/neu*].

## Introduction

The anthracycline class chemotherapeutics intercalate between DNA strands to exert their mechanism-of-action that in turn inhibits DNA and RNA synthesis in addition to triggers topoisomerasae II mediated DNA cleavage resulting in the promotion of cell death. Binding to cell membranes and plasma proteins may also be involved in the cytotoxic properties of the anthracyclines which is complemented by outright injury to neoplastic cells secondary to the generation of highly reactive free radicals species. Each of these mechanisms-of-action collectively promotes apoptosis. In their clinical usage, the anthracycline are among the most potent and clinically effective class of chemotherapeutics for the treatment of mammary carcinoma, ovarian carcinoma, colon carcinoma, and acute myeloid leukemia.

Gemcitabine is a deoxycytadine nucleotide analog that intra-cellularly has a mechanism-of-action that involves it being triphosphoralated in a manner that allows it to substitute for cytadine during DNA transcription resulting in incorporation into DNA strands and inhibit the biochemical activity of DNA polymerase. A second mechanism-of-action for gemcitabine involves inhibition and inactivation of ribonucleotide reductase and ultimately the suppression of deoxyribonucleotide synthesis in concert with diminished DNA repair and reduced DNA transcription. Each of these mechanisms-of-action collectively promotes cellular apoptosis. Features of the pharmacokinetic profile for gemcitabine include a brief plasma half-life because it is rapidly deaminated to an inactive metabolite that is rapidly eliminated through renal excretion into the urine[1–3].

In clinical oncology, the anthracycline chemotherapeutics are commonly administered to treat breast cancer and many other neoplastic conditions due to their superior level of potency. Gemcitabine is administered for the treatment certain leukemias and potentially lymphoma conditions in addition to a spectrum of adenocarcinomas and carcinomas affecting the lung (e.g. non-small cell), pancreas, urinary bladder and esophagus. Gemcitabine has a brief plasma half-life because it is rapidly deaminated to an inactive metabolite that is rapidly eliminated through renal excretion into the urine[1–3]. Despite their superior clinical effectiveness in modern clinical oncology, the anthracyclines, gemcitabine, and many other chemotherapeutic agents often have relatively low margins-of-safety largely because almost invariably they impose a high risk for inducing serious sequelae especially when administered as a component of a long-term treatment regimen. The most common and dose-limiting side effect of anthracycline administration is cardiotoxicity which is more prominent with doxorubicin compared to epirubicin which is excreted more rapidly than doxorubicin presumably due to a difference in the spatial orientation of the hydroxyl (-OH) group at the C4-carbon of the carbohydrate-like moiety.

Although the anthracyclines and gemcitabine exert high levels of anti-neoplastic cytotoxicity, when applied as a monotherapy they are still usually incapable of completely resolving most types of neoplastic disease such as resistant and aggressive forms of breast cancer. Mono-therapy treatment regimens also pose a higher risk chemotherapeutic-resistance in neoplastic cell populations which is a confounding variable that can either be induced de-novo or acquired through selective pressure. Transformations of neoplastic cells of this type has many implications in clinical oncology for the management breast cancer where 20–30% of all affected cases develop metastatic brain lesions that characteristically display moderate-to-high levels of resistance to chemotherapeutic intervention[4]. Combination chemotherapeutic regimens are almost invariably more potent and effective in suppressing growth and metastasis, delaying the onset of disease relapse, prolonging the onset of disease remission, and improving the probability of complete neoplastic disease elimination. Despite the advantages of combination regimens, anytime conventional chemotherapeutics are administered *in-vivo* in “free form” they still pose a high risk frequency for toxic sequelae that can ultimately limit the extent and duration of therapeutic intervention[5–14].

Alternative “newer generation” treatment modalities such as monoclonal immunoglobulin that inhibit the function of trophic receptor complexes uniquely or highly over-expressed by populations of a given neoplastic cell type offer an opportunity for avoiding many of the common side effects associated with conventional chemotherapeutics. Monoclonal immunoglobulin fractions with binding-avidity for trophic membrane receptors that are over-expressed by neoplastic cell types including HER2/*neu* (e.g. anti-HER2/*neu*: trastuzumab, pertuzumab),[15–19] EGFR (e.g. anti-EGFR: cetuximab, gefitinib), [20–23] both HER2/*neu* and EGFR (e.g. anti-HER2/*neu* and anti-EGFR: panitumumab),[22–25] and IGFR (e.g. figitumumab, dalotuzumab)[26–29] can all be effective treatment options for cancer including forms of neoplasia affecting the breast, intestinal tract, lung and prostate. One obvious advantage of these preparations is their ability to function as anti-cancer treatment modalities that avoid many of the sequelae associated with conventional chemotherapeutics. Unfortunately, most monoclonal immunoglobulin-based therapies that inhibit the function of trophic membrane receptors are usually only capable of exerting cytostatic properties and are almost invariably plagued by an inability to evoke cytotoxic activity sufficient to independently resolve most aggressive or advanced forms of neoplastic disease.[15,16,30–44] Increases in cell-cycle G_1_-arrest, cellular transformation to states of apoptosis-resistance,[31] and selection for resistant sub-populations[15,16] in part are a refection of the lack of cytotoxic efficacy of anti-trophic receptor immunoglobulins that can be further complicated by frequent reversal of tumor growth inhibition[15] and relapse trophic receptor over-expression[30] following therapeutic withdrawl. However, additive or synergistic levels of anti-neoplastic potency can be attained with anti-trophic receptor immunoglobulin fractions when they are applied in dual-combination with conventional chemotherapeutics. [45–47] Inhibition of HER2/*neu* function with anti-HER2/*neu* results in enhanced levels of anti-neoplastic cytotoxicity when it is applied in concert with cyclophosph*amide*,[46,48] docetaxel,[48] doxorubicin,[46;48] etoposide,[48] methotrexate,[48] paclitaxel,[46,48] or vinblastine.[48] Similar to anti-HER2/*neu*,[46,48–52] other trophic receptor site inhibitors including anti-EGFR,[53–55] anti-IGFR-1,[56,57] and anti-VEGFR[45,58,59] also create additive and synergistic levels of anti-neoplastic cytotoxicity when applied in combination with conventional chemotherapeutic agents.

Covalent immunochemotherapeutics that possess properties of selective “targeted” delivery have traditionally been synthesized utilizing the anthracyclines[60–85]. where doxorubicin[86–90] has been most commonly been utilized to date, and to a lesser extent, daunorubicin[91–93] and epirubicin[66,85,94,95]. Covalent immunochemotherapeutics of this type utilize monoclonal immunoglobulin fractions, and occasionally receptor ligands, receptor ligand fragments or synthetic ligands that recognize and physically bind to specific antigens or receptor complexes uniquely over-expressed on the exterior surface membrane of neoplastic cell populations[66,85,88,89,96,97]. Gemcitabine chemotherapeutic has been covalently bonded to large molecular weight platforms much less frequently compared to the anthracyclines and a very limited number of published reports have described the synthesis and anti-neoplastic cytotoxicity of covalent gemcitabine immunochemotherapeutics capable of facilitating selective “targeted” delivery[97–99]. Despite rather extensive familiarity with biological effect of anti-HER2/*neu* and anti-EGFR on the vitality of cancer cell populations and it’s application in clinical oncology, there has correspondingly been surprisingly little research devoted to the molecular design, chemical synthesis and potency evaluation of covalent anthracycline and especially gemcitabine immunochemotherapeutics[97]. Even less knowledge currently exists about the potential for dual covalent immunochemotherapeutic combinations to additively and synergistically attain enhanced levels of anti-neoplastic cytotoxicity[97]. Given this perspective, gemcitabine-(C_4_-*amide*)-[anti-EGFR] and epirubicin-(C_3_-*amide*)-[anti-HER2/*neu*] were applied simultaneously in a dual-combination to detect their potential to evoke additive or synergistic levels of anti-neoplastic cytotoxicity against chemotherapeutic-resistant mammary adenocarcinoma (SKBr-3). Complementary investigations delineated the potential for benzimidazole tubulin/microtubule inhibitors to complement the anti-neoplastic cytotoxicity of gemcitabine-(C_4_-*amide*)-[anti-EGFR] applied in dual-combination with epirubicin-(C_3_-*amide*)-[anti-HER2/*neu*]. Investigations ultimately demonstrated how gemcitabine-(C_4_-*amide*)-[anti-EGFR] and epirubicin-(C_3_-*amide*)-[anti-HER2/*neu*] can selectively “target” the delivery of two different chemotherapeutic agents at two different unique or over-expressed receptors over-expressed by neoplastic cell types. The anti-neoplastic cytotoxicity of gemcitabine-(C_4_-*amide*)-[anti-EGFR] and epirubicin-(C_3_-*amide*)-[anti-HER2/*neu*] is complemented by the biological activity of benzimidazole tubulin/microtubule inhibitors.

## Materials and Methods

### Covalent gemcitabine and epirubicin immunochemotherapeutic synthesis

**Figure 1:**
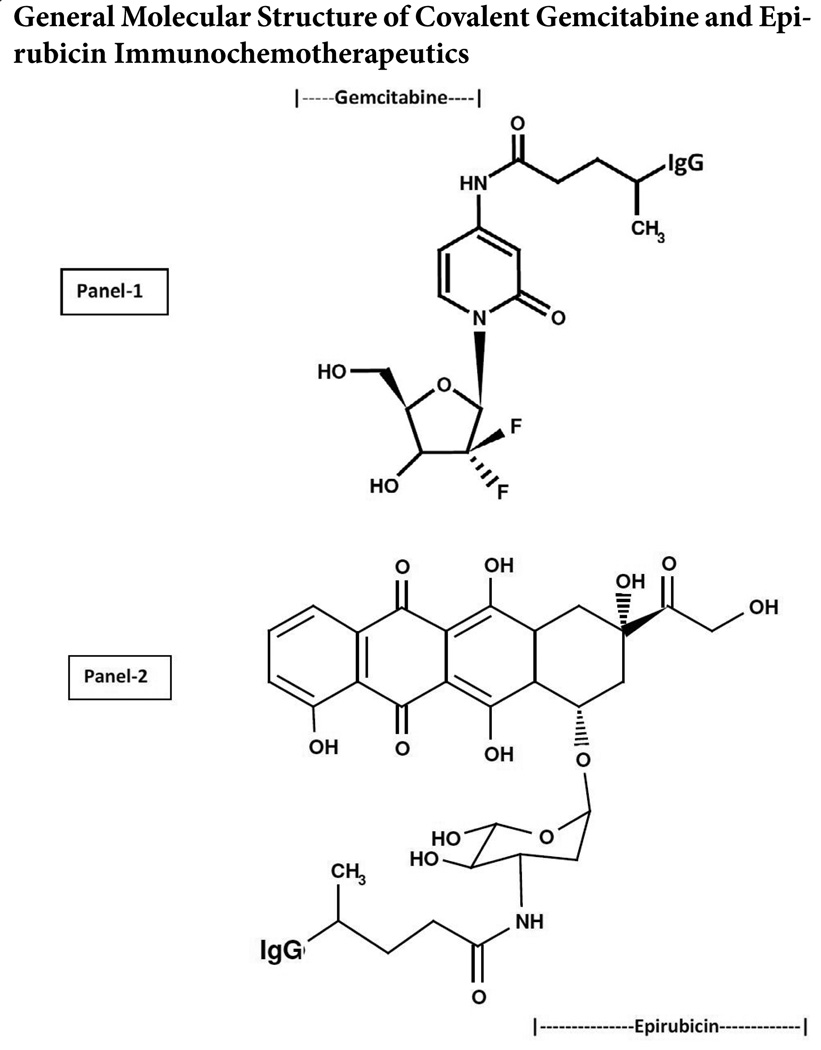
Molecular design and chemical composition of covalent gemcitabine and epirubicin immunochemotherapeutics (Panel 1) gemcitabine-(C_4_-*amide*)-[anti-EGFR]; and (Panel 2) epirubicin-(C_3_-*amide*)-[anti-HER2/*neu*]. Both covalent immunochemotherapeutics were synthesized utilizing a 2-stage organic chemistry reaction scheme that initially produces a chemotherapeutic analog that is a UV-photoactivated intermediate. Covalent bonds are formed at the mono-amine groups of gemcitabine or epirubicin and the side chains of amino acid residues within the sequence of immunoglobulin fractions.

Phase-I Synthesis Scheme for UV-Photoactivated Chemotherapeutic Intermediates- The cytosine-like C_4_-amine of gemcitabine (0.738mg, 2.80 × 10^−3^mMoles) or the C_3_ α-monoamine on the carbohydrate-type moiety of epirubicin was reacted at a 2.5:1 molar-ratio with the amine-reactive N-hydroxysuccinimide ester “leaving” complex of succinimidyl 4,4-azipentanoate (0.252mg, 1.12 × 10^−3^mMoles) in the presence of triethylamine (TEA: 50mM final concentration) utilizing dimethylsulfoxide as an anhydrous organic solvent system (Figure. 1). Formulated from stock solutions, the reaction mixture containing gemcitabine and succinimidyl 4,4-azipentanoate, or epirubicin and succinimidyl 4,4-azipentanoate was continually stirred gently at 25° C over a 4-hour incubation period in the dark and protected from exposure to light. The relatively long incubation period of 4 hours was utilized to maximize degradation of the ester group associated with any residual succinimidyl 4,4-azipentanoate that may not of reacted during the first 30 to 60 minutes with the C_4_ cytosine-like mono-amine group of gemcitabine or the C_3_ α-monoamine of the epirubicin carbohydrate-type moiety.

Phase-II Synthesis Scheme for Covalent Gemcitabine and Epirubicin Immunochemotherapeutics Utilizing a UV-Photoactivated Chemotherapeutic Intermediate- Immunoglobulin fractions of anti-HER2/*neu* or anti-EGFR (1.5mg, 1.0 × 10^−5^mMoles) in buffer (PBS: phosphate 0.1, NaCl 0.15M, EDTA 10mM, pH 7.3) were combined at a 1:10 molar-ratio with either the UV-photoactivated gemcitabine-(C_4_-*amide*) or epirubicin-(C_3_-*amide*) intermediate (Phase-1 end product) and were initially allowed to gently mix by constant stirring for 5 minutes at 25° C in the dark. The photoactivated group of the gemcitabine-(C_4_-*amide*) or epirubicin-(C_3_-*amide*) reactive intermediates was covalently bonded to chemical groups associated with sides chains of amino acid residues in the sequence of anti-EGFR or anti-HER2/*neu* monoclonal immunoglobulin fractions during a 15 minute exposure to UV light at 354 nm (reagent activation range 320–370 nm) in combination with constant gentle stirring (Figure. 1). Residual un-reacted (“free” non-protein associated) gemcitabine or epirubicin was removed from covalent immunochemotherapeutic micro-scale column chromatography following pre-equilibration of exchange media with PBS (phosphate 0.1M, NaCl 0.15M, pH 7.3).

### Molecular analysis and characterization of properties

#### General analysis

Quantification of the amount of non-covalently bound gemcitabine or epirubicin contained within gemcitabine-(C_4_-*amide*)-[anti-EGFR] and epirubicin-(C_3_-*amide*)-[anti-HER2/*neu*] preparations respectively entailed initial protein precipitation of the covalent immunochemotherapeutics with methanol:acetonitrile (1:9 v/v) and subsequent measurement of gemcitabine (absorbance: 265–268nm),[98,100,101] or epirubicin (Ex/Em: 485nm/538nm) [17,66,102] in the resulting supernatant.

Quantification of the amount of covalently bound gemcitabine or epirubicin was performed at previously described for gemcitabine-(C_4_-*amide*)-[anti-EGFR][97–99] and epirubicin-(C_3_-*amide*)-[anti-HER2/*neu*][66,85,102]. Measurements from these analyses were utilized to calculate the gemcitabine and epirubicin molar-incorporation-indexes for gemcitabine-(C_4_-*amide*)-[anti-EGFR] and epirubicin-(C_3_-*amide*)-[anti-HER2/*neu*].

Determination of the immunoglobulin concentration for the covalent gemcitabine-(C_4_-*amide*)-[anti-EGFR] and epirubicin-(C_3_-*amide*)-[anti-HER2/*neu*] immunochemotherapeutics was determined by measuring absorbance at 280nm in combinations with utilizing a 235nm -vs- 280nm standardized reference curve in order to accommodate for any potential absorption profile over-lap at 280nm between immunoglobulin and the chemotherapeutic moieties of gemcitabine and epirubicin.

#### Mass/size-dependent separation of gemcitabine-immuno-chemotherapeutics by non-reducing SDS-PAGE

Covalent gemcitabine-(C_4_-*amide*)-[anti-EGFR] and epirubicin-(C_3_-*amide*)-[anti-HER2/*neu*] immunochemotherapeutics in addition to reference control anti-EGFR and anti-HER2/*neu* immunoglobulin fractions were adjusted to a standardized protein concentration of 60µg/ml and then combined 50/50 v/v with conventional SDS-PAGE sample preparation buffer (Tris/glycerol/bromphenyl blue/SDS) formulated without 2-mercaptoethanol or boiling. Each covalent immunochemotherapeutic, the reference control immunoglobulin fraction (0.9µg/well) and a mixture of pre-stained reference control molecular weight markers were then developed by non-reducing SDS-PAGE (11% acryl*amide*) performed at 100 V constant voltage at 3°C for 2.5 hours.

#### Western-blot immunodetection analyses

Covalent gemcitabine-(C_4_-*amide*)-[anti-EGFR] and epirubicin-(C_3_-*amide*)-[anti-HER2/*neu*] immunochemotherapeutics following mass/size-dependent separation by non-reducing SDS-PAGE were equilibrated in tank buffer devoid of methanol. Mass/size-separated gemcitabine-(C_4_-*amide*)-[anti-EGFR] and epirubicin-(C_3_-*amide*)-[anti-HER2/*neu*] contained in acryl*amide* SDS-PAGE gels were then transferred laterally onto sheets of nitrocellulose membrane at 20 volts (constant voltage) for 16 hours at 2° to 3°C with the transfer manifold packed in crushed ice.

Nitrocellulose membranes with laterally-transferred immunochemotherapeutics were then equilibrated in Tris buffered saline (TBS: Tris HCl 0.1M, NaCl 150mM, pH 7.5, 40ml) at 4°C for 15 minutes followed by incubation in TBS blocking buffer solution (Tris 0.1M, pH 7.4, 40ml) containing bovine serum albumin (5%) for 16 hours at 2 for 16 hours at 2° to 3°C applied in combination with gentle horizontal agitation. Prior to further processing, nitrocellulose membranes were vigorously rinsed in Tris buffered saline (Tris 0.1M, pH 7.4, 40ml, n = 3×).

Nitrocellulose membranes following BSA-block and serial rinsing were then incubated with biotinylated goat anti-murine IgG (1:10,000 dilution) at 4°C for 18 hours applied in combination with gentle horizontal agitation. Nitrocellulose membranes were then vigorously rinsed in TBS (pH 7.4, 4° C, 50ml, n = 3) followed by incubation in blocking buffer (Tris 0.1M, pH 7.4, with BSA 5%, 40ml). Blocking buffer was decanted from nitrocellulose membrane blots which were then rinsed in TBS (pH 7.4, 4° C, 50ml, n = 3) before incubation with strepavidin-HRPO (1:100,000 dilution) at 4°C for 2 hours applied in combination with gentle horizontal agitation. Prior to chemiluminescent development nitrocellulose membranes were vigorously rinsed in Tris buffered saline (Tris 0.1M, pH 7.4, 40ml, n = 3). Following development with conjugated HR-PO-strepavidin each nitrocellulose membrane was then incubated with HRPO chemiluminescent substrate (25°C; 5-to-10 minutes). Chemiluminescent autoradiography images were acquired by exposing radiographic film (Kodak BioMax XAR) to nitrocellulose membranes sealed within transparent ultra-clear re-sealable plastic bags.

### Mammary adenocarcinoma: Neoplastic disease *ex-vivo* model

#### Mammary adenocarcinoma tissue culture cell culture

The human mammary adenocarcinoma (SKBr-3) was utilized as an *ex-vivo* model for neoplastic disease. Populations of the mammary adenocarcinoma (SKBr-3) were propagated at >85% level of confluency in 150-cc^2^ tissue culture flasks containing McCoy's 5a Modified Medium supplemented with fetal bovine serum (10% v/v) and penicillin-streptomycin at a temperature of 37° C under a gas atmosphere of air (95%) and carbon dioxide (5% CO_2_). Trypsin or any other biochemically active enzyme fraction were not used to facilitate harvest of mammary adenocarcinoma SKBr-3 cell suspensions for seeding of tissue culture flasks or multi-well tissue culture plates. Growth media was not supplemented with growth factors, growth hormones or any other type of growth stimulant. Characteristic features and biological properties of the mammary adenocarcinoma (SKBr-3) cell line includes chemotherapeutic-resistance, overexpression of epidermal growth factor receptor 1 (EGFR, ErbB-1, HER1: at 2.2 × 10^5^/cell), and high over-expression of epidermal growth factor receptor 2 (EGFR2, HER2/*neu*, ErbB-2, CD340, p185: at 1 × 10^6^/cell).

#### Cell-ELISA total membrane-bound immunoglobulin assay

Cell suspensions of mammary adenocarcinoma (SKBr-3) were seeded into 96-well microtiter plates in aliquots of 2 × 10^5^ cells/well and allowed to form a confluent adherent monolayer over a period of 48 hours. The growth media content of each individual well was removed manually by pipette and cellular monolayers were then serially rinsed (n = 3) with PBS followed by their stabilization onto the plastic surface of 96-well plates with paraformaldehyde (4% in PBS, 15 minutes). Stabilized cellular monolayers were then incubated with covalent gemcitabine-(C_4_-*amide*)-[anti-EGFR] and epirubicin-(C_3_-*amide*)-[anti-HER2/*neu*] immunochemotherapeutics formulated at gradient concentrations of 0.1, 0.25, 0.5, 1.0, 5.0 and 10µg/ml in tissue culture growth media (200µl/well). Direct contact incubation between (SKBr-3) cellular monolayers and gemcitabine-(C_4_-*amide*)-[anti-EGFR] and epirubicin-(C_3_-*amide*)-[anti-HER2/*neu*] was performed at 37° C over an incubation period of 3-hours using a gas atmosphere of air (95%) and carbon dioxide (5% CO_2_). Following serial rinsing with PBS (n = 3), development of stabilized mammary adenocarcinoma (SKBr-3) monolayers entailed incubation with β-galactosidase conjugated goat anti-mouse IgG (1:500 dilution) for 2 hours at 25° C with residual unbound immunoglobulin removed by serial rinsing with PBS (n = 3). Final cell ELISA development required serial rinsing (n = 3) of stabilized (SKBr-3) monolayers with PBS followed by incubation with nitrophenyl-β-D-galactopyranoside substrate (100µl/well of ONPG formulated fresh at 0.9 mg/ml in PBS pH 7.2 containing MgCl_2_ 10mM, and 2-mercaptoethanol 0.1M). Absorbance within each individual well was measured at 410nm (630nm reference wavelength) after incubation at 37° C for a period of 15 minutes.

#### Anti-neoplastic cytotoxicity

Individual preparations of gemcitabine-(C_4_-*amide*)-[anti-EGFR] and epirubicin-(C_3_-*amide*)-[anti-HER2/*neu*] were formulated in growth media at standardized chemotherapeutic-equivalent concentrations of 10^−10^, 10^−9^, 10^−8^, 10^−7^, and 10^−6^ M (final concentration). Each chemotherapeutic-equivalent concentration of covalent immunochemotherapeutic was then transferred in triplicate into 96-well microtiter plates containing mammary adenocarcinoma (SKBr-3) monolayers and growth media (200µl/well). Covalent immunochemotherapeutics where then incubated in direct contact with monolayer populations of mammary adenocarcinoma (SKBr-3) for a period of 182-hours (37° C under a gas atmosphere of air (95%) and carbon dioxide/CO_2_ (5%). Following the initial 96-hour incubation period, mammary adenocarcinoma (SKBr-3) populations were replenished with fresh tissue culture media with or without covalent immunochemotherapeutics or benzimidazole tubulin/microtubule inhibitors.

Cytotoxic potencies for gemcitabine-(C_4_-*amide*)-[anti-EGFR] and epirubicin-(C_3_-*amide*)-[anti-HER2/*neu*] were measured by removing all contents within the 96-well microtiter plates manually by pipette followed by serial rinsing of monolayers (n = 3) with PBS and incubation with 3-[4,5-dimethylthiazol-2-yl]-2,5-diphenyl tetrazolium bromide vitality stain reagent formulated in RPMI-1640 growth media devoid of pH indicator or bovine fetal calf serum (MTT: 5mg/ml). During an incubation period of 3–4 hours at 37° C under a gas atmosphere of air (95%) and carbon dioxide (5% CO_2_) the enzyme mitochondrial succinate dehydrogenase was allowed to convert the MTT vitality stain reagent to navy-blue formazone crystals within the cytosol of mammary adenocarcinoma (SKBr-3) populations (some reports suggest that NADH/NADPH dependent cellular oxidoreductase enzymes may also be involved in the biochemical conversion process). Contents of the 96-well microtiter plate was then removed, followed by serial rinsing with PBS (n = 3). The resulting blue intracellular formazone crystals were dissolved with DMSO (300µl/well) and then the spectrophotometric absorbance of the blue-colored supernatant measured at 570nm using a computer-integrated microtiter plate reader.

## Results

### Molar-incorporation index

Size-separation of gemcitabine-(C_4_-*amide*)-[anti-EGFR] and epirubicin-(C_3_-*amide*)-[anti-HER2/*neu*] by micro-scale desalting/buffer exchange column chromatography consistently yields covalent immunochemotherapeutic preparations that contained <4.0% of residual chemotherapeutic that was not covalently bound to immunoglobulin[66,85,97,98,102]. Small residual amounts of non-covalently bound chemotherapeutic remaining within covalent immunochemotherapeutic preparations is generally accepted to not be available for further removal through any additional sequential column chromatography separations.[103] The calculated estimate of the molar-incorporation-index for the covalent gemcitabine-(C_4_-*amide*)-[anti-HER2/*neu*] immunochemotherapeutic was 2.78 utilizing the organic chemistry reaction scheme to form an *amide* bond at the cytosine-like mono-amine of gemcitabine and synthesis of the UV-photoactivated gemcitabine-(C_4_-*amide*) intermediate (Figure. 1). The molar-incorporation-ration of 2.78-to-1 for gemcitabine-(C_4_-*amide*)-[anti-HER2/*neu*] was relatively larger than the 1.1-to-1 gemcitabine molar-incorporation-index attained during the synthesis of gemcitabine-(C5-methylcarbamate)-[anti-HER2/*neu*][97].

### Molecular weight profile analysis

**Figure 2:**
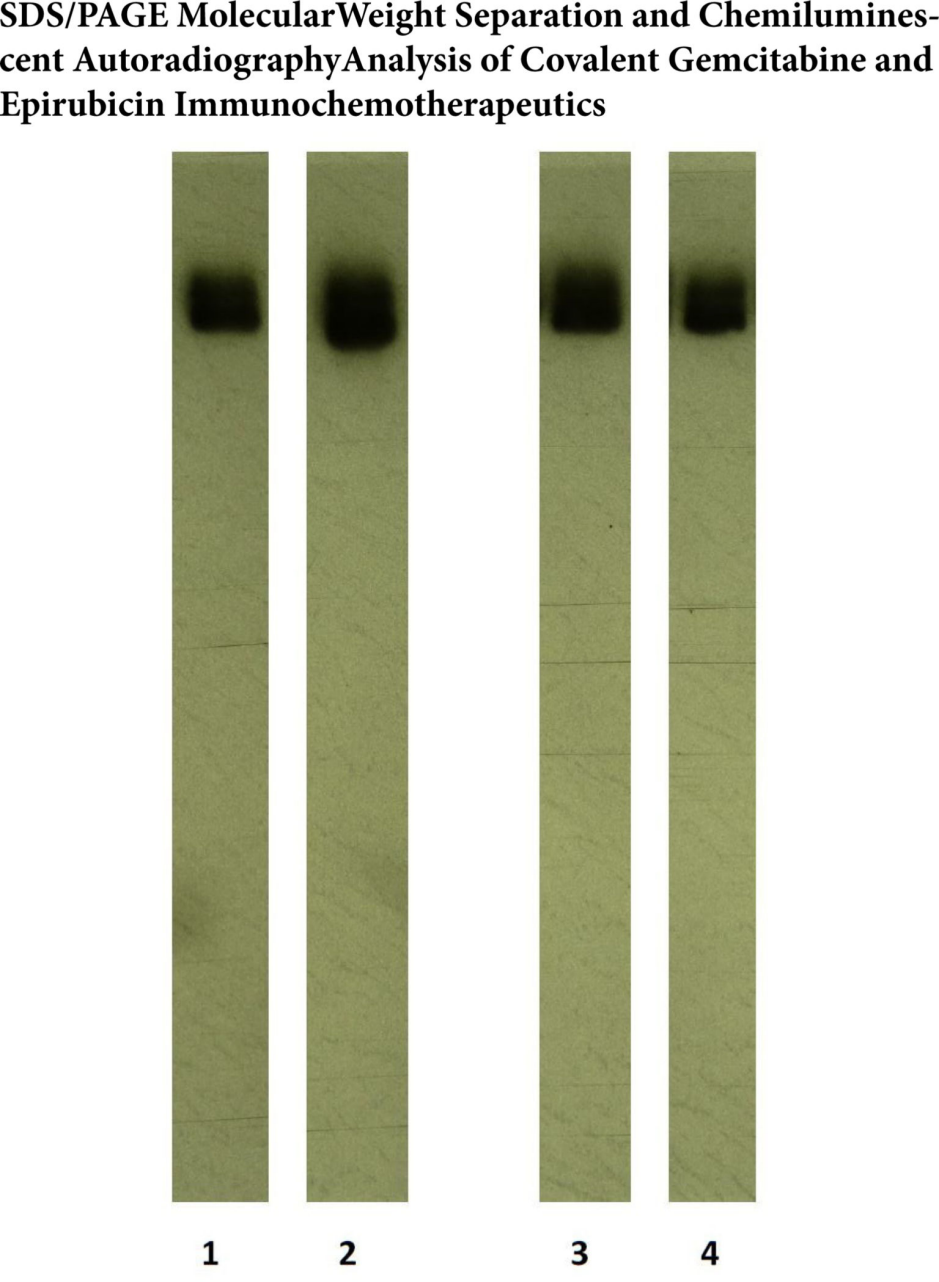
Characterization of the molecular weight profile for the covalent immunochemotherapeutics, epirubicin-(C_3_-*amide*)-[anti-HER2/*neu*] and gemcitabine-(C_4_-*amide*)-[anti-EGFR] relative to reference control anti-EGFR and anti-HER2/*neu* monoclonal immunoglobulin fractions (Lane-1) murine anti-human EGFR monoclonal immunoglobulin; (Lane-2) gemcitabine-(C_4_-*amide*)-[anti-EGFR]; (Lane-3) murine anti-human HER2/*neu* monoclonal immunoglobulin; and (Lane-4) epirubicin-(C_3_-*amide*)-[anti-HER2/*neu*]; Covalent immunochemotherapeutics and monoclonal immunoglobulin fractions were size-separated by non-reducing SDS-PAGE followed by lateral transfer onto sheets of nitrocellulose membrane to facilitate detection with biotinylated goat anti-mouse IgG immunoglobulin. Subsequent analysis entailed incubation of membranes with strepavidin-HRPO in combination with the use of a HRPO chemiluminescent substrate and the acquisition of autoradiography images.

Mass/size separation of covalent gemcitabine-(C_4_-*amide*)-[anti-EGFR] and epirubicin-(C_3_-*amide*)-[anti-HER2/*neu*] immunochemotherapeutics by SDS-PAGE in combination with immunodetection analysis (Western blot) and chemiluminescent autoradiography recognized a single primary condensed band of 150-kDa between a molecular weight range of 5.0-kDa to 450-kDa (Figure. 2) Patterns of low-molecular-weight fragmentation (proteolytic/hydrolytic degradation) or large-molecular-weight immunoglobulin polymerization were not detected (Figure. 2). The observed molecular weight of 150-kDa for both gemcitabine-(C_4_-*amide*)-[anti-EGFR] and epirubicin-(C_3_-*amide*)-[anti-HER2/*neu*] directly corresponds with the known molecular weight/mass of reference control anti-HER2/*neu* monoclonal immunoglobulin fractions (Figure. 2). Analogous results have been reported for similar covalent immunochemotherapeutics[61,66,85,97,98,102,104].

### Cell-Binding Analysis

**Figure 3:**
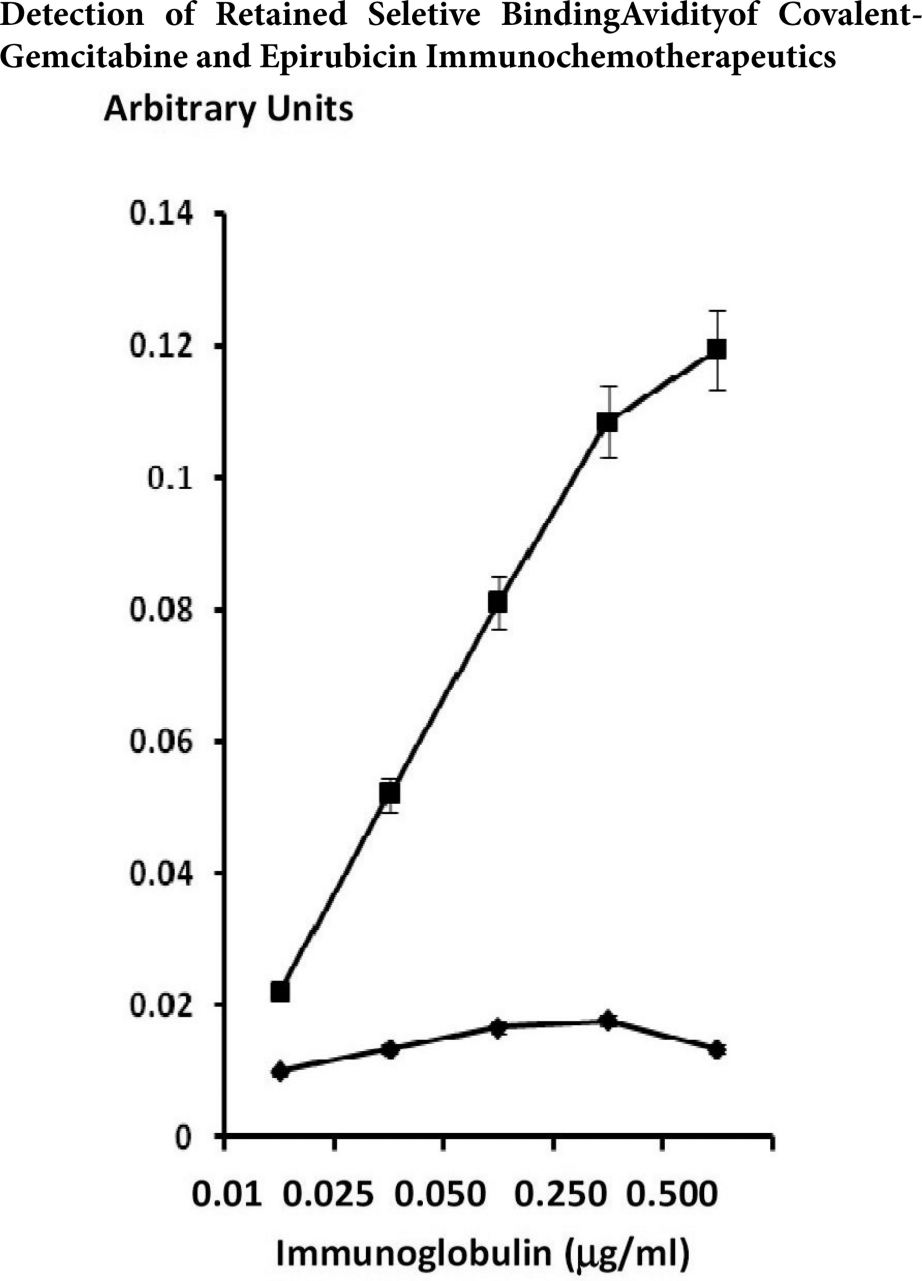
Detection of total immunoglobulin in the form of gemcitabine-(C_4_-*amide*)-[anti-EGFR] or epirubicin-(C_3_-*amide*)-[anti-HER2/*neu*] selectively bound to the exterior surface membrane of chemotherapeutic-resistant mammary adenocarcinoma (◆) gemcitabine-(C_4_*amide*)-[anti-EGFR]; and (■) epirubicin-(C_3_-*amide*)-[anti-HER2/*neu*]. Covalent gemcitabine-(C_4_-*amide*)-[anti-EGFR] or epirubicin-(C_3_-*amide*)-[anti-HER2/*neu*] immunochemotherapeutics formulated at gradient concentrations were incubated with triplicate monolayer populations of chemotherapeutic-resistant mammary adenocarcinoma (SKBr-3) over a 4-hour period and total immunoglobulin bound to the exterior surface membrane was then measured by cell-ELISA.

Total bound immunoglobulin in the form of gemcitabine-(C_4_-*amide*)-[anti-EGFR] and epirubicin-(C_3_-*amide*)-[anti-HER2/*neu*] on the external surface membrane of adherent mammary adenocarcinoma (SKBr-3) populations was measured by cell-ELISA (Figure. 3). Greater total membrane-bound gemcitabine-(C_4_-*amide*)-[anti-EGFR] and epirubicin-(C_3_-*amide*)-[anti-HER2/*neu*] was detected with progressive increases in standardized total immunoglobulin-equivalent concentrations formulated at 0.010, 0.025, 0.050, 0.250, and 0.500µg/ml (Figure. 3). Collectively each of these sets of cell-ELISA findings serve to validate the retained selective binding-avidity of gemcitabine-(C4-*amide*)-[anti-EGFR] and epirubicin-(C_3_-*amide*)-[anti-HER2/*neu*] for over-expressed EGFR (2.2 × 10^5^ per cell) and highly over-expressed HER2/*neu* (1 × 10^6^ per cell) trophic receptor sites on the exterior surface membrane of mammary adenocarcinoma (SKBr-3) populations (Figure. 3)[97].

### Anti-neoplastic cytotoxicity

**Figure 4:**
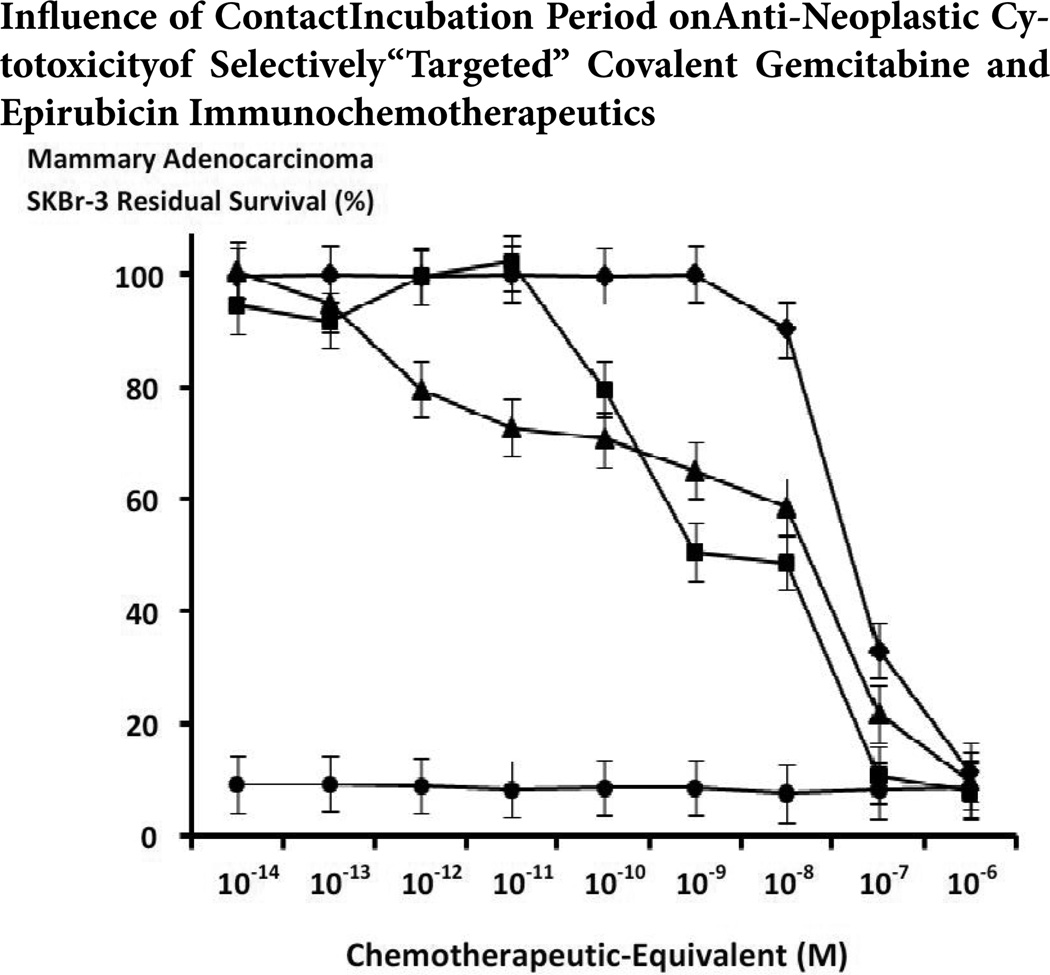
Relative anti-neoplastic cytotoxicity for the covalent immunochemotherapeutic dual-combination of gemcitabine-(C_4_-*amide*)-[anti EGFR] and epirubicin-(C_3_-*amide*)-[anti-HER2/*neu*] compared to gemcitabine-(C_4_-*amide*)-[anti-EGFR] alone against chemotherapeutic-resistant human mammary adenocarcinoma (■) gemcitabine-(C_4_-*amide*)-[anti-EGFR] in dual-combination with epirubicin-(C_3_-*amide*)-[anti-HER2/*neu*] at 182-hours; (▲) gemcitabine-(C_4_-*amide*)-[anti-EGFR] at 182-hours; and (◆) epirubicin-(C_3_-*amide*)-[anti-HER2/*neu*] at 96-hours; (●) epirubicin-(C_3_-*amide*)-[anti HER2/*neu*] at 182-hours. Individual covalent immunochemotherapeutic or the dual 50/50 combination of gemcitabine-(C_4_-*amide*)-[anti-EGFR with epirubicin-(C_3_-*amide*)-[anti-HER2/*neu*] were formulated at gradient chemotherapeutic-equivalent concentrations and incubated in direct con tact with triplicate monolayer populations of chemotherapeutic-resistant mammary adenocarcinoma (SKBr-3) for period of 182-hours. Anti-neo plastic cytotoxicity was detected and measured using a MTT cell vitality assay and values reported as a percentage of matched negative reference controls (100%).

Anti-neoplastic cytotoxicity of gemcitabine-(C_4_-*amide*)-[anti-EGFR] after 182-hours was consistently greater than epirubicin-(C_3_-*amide*)-[anti-HER2/*neu*] following a 96-hour incubation period against chemotherapeutic-resistant mammary adenocarcinoma (SKBr-3) at and between chemotherapeutic-equivalent concentrations of 10^−13^ M and 10^−7^ M (Figure. 4). Based on the difference in contact incubation periods applied and the levels of anti-neoplastic potency acquired, it can be assumed that epirubicin-(C_3_-*amide*)-[anti-HER2/*neu*] is relatively more potent than gemcitabine-(C_4_-*amide*)-[anti-EGFR] over a direct contact incubation period of 96-hours (Figure. 4). Gemcitabine-(C_4_-*amide*)-[anti-EGFR] produced progressively higher levels of anti-neoplastic cytotoxicity of 0.0% at 10^−14^ M (100% residual survival) to 41.4% at 10^−8^ M (58.6% residual survival) followed by a relatively more rapid increase from 41.4% to 90.1% at and between 10^−8^ M and 10^−6^ M (58.6% and 9.86% residual survival) respectively (Figure. 4). Alternatively, relatively rapid increases in anti-neoplastic cytotoxicity from 0.0% to 88.5% were detected for epirubicin-(C_3_-*amide*)-[anti-R2/*neu*] at and between chemotherapeutic-equivalent concentrations of 10^−9^ M and 10^−6^ M (100% to 11.5% residual survival) respectively (Figure. 4). Epirubicin-(C_3_-*amide*)-[anti-HER2/*neu*] following a 182-hour incubation period produced essentially identical levels of anti-neoplastic cytotoxicity that varied between 7.7% and 9.3% residual survival for chemotherapeutic-equivalent concentrations at and between 10^−14^ M and 10^−6^ M (Figure. 4).

**Figure 5:**
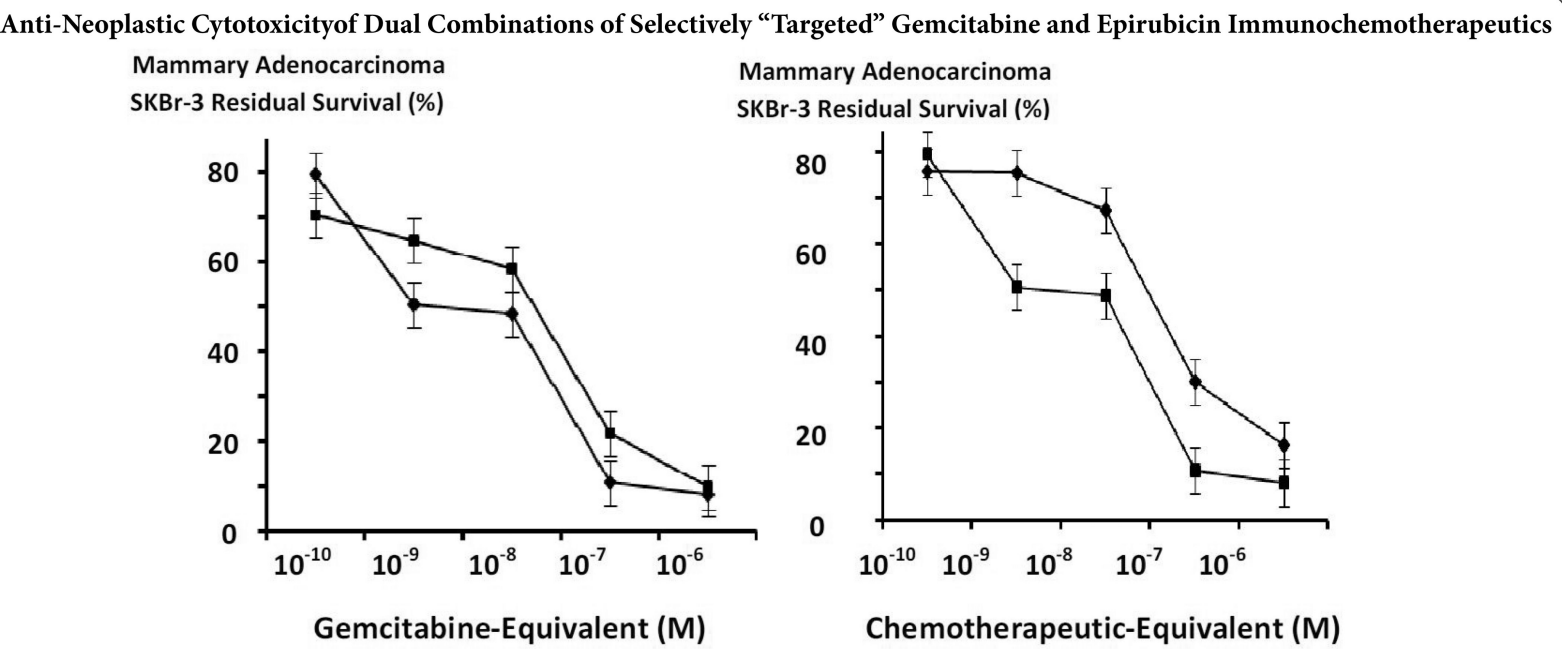
Relative anti-neoplastic cytotoxicity for the dual-combination of covalent gemcitabine and epirubicin immunochemotherapeutics against chemotherapeutic-resistant human mammary adenocarcinoma Left-Panel (■) gemcitabine-(C_4_-*amide*)-[anti-EGFR], and (◆) gemcitabine-(C_4_-*amide*)-[anti-EGFR] with epirubicin-(C_3_-*amide*)-[anti-HER2/*neu*]. Right-Panel (■) gemcitabine-(C_4_-*amide*)-[anti-EGFR] with epirubicin-(C_3_-*amide*)-[anti-HER2/*neu*]; and (◆) gemcitabine-(C_4_-*amide*)-[anti-EGFR] with gemcit-abine-(C_4_-*amide*)-[anti-HER2/*neu*]. Dual-combinations of covalent immunochemotherapeutics were formulated at gradient 50/50 chemotherapeutic-equivalent concentrations. Both individual and dual covalent immunochemotherapeutic combinations were incubated in direct contact with triplicate monolayer populations of chemotherapeutic-resistant mammary adenocarcinoma (SKBr-3) over a period of 182-hours. Anti-neoplastic cytotoxicity was detected and measured using a MTT cell vitality assay relative to matched negative reference controls.

The anti-neoplastic cytotoxicity profiles for gemcitabine-(C_4_-*amide*)-[anti-EGFR] in dual-combination with epirubicin-(C_3_-*amide*)-[anti-HER2/*neu*] compared to gemcitabine-(C_4_-*amide*)-[anti-EGFR] alone were somewhat similar. Rapid progressive increases in anti-neoplastic cytotoxicity for gemcitabine-(C_4_-*amide*)-[anti-EGFR] in dual-combination with epirubicin-(C_3_-*amide*)-[anti-HER2/*neu*] from 0.0% to 91.9% were detected at and between the chemotherapeutic-equivalent concentrations of 10^−11^ M and 10^−6^ M (100% and 8.1% residual survival) respectively (Figures. 4 and 5). Alternatively, gemcitabine-(C_4_-*amide*)-[anti-EGFR] created progressive and substantial increases in anti-neoplastic cytotoxicity from 0.0% to 90.1% at and between the chemotherapeutic-equivalent concentrations of 10^−14^ M and 10^−6^ M (100% to 9.9% residual survival) respectively (Figures. 4 and 5). Levels of anti-neoplastic cytotoxicity for gemcitabine-(C_4_-*amide*)-[anti-EGFR] in dual-combination with epirubicin-(C_3_-*amide*)-[anti-HER2/*neu*] were very similar to gemcitabine-(C_4_-*amide*)-[anti-EG-FR] at and between the chemotherapeutic concentrations of 10^−10^ M (79.5% -vs- 70.6% residual survival) and the maximum concentration of 10^−6^ M (8.1% -vs- 9.9% residual survival) respectively (Figures. 4 and 5).

The anti-neoplastic cytotoxicity profiles for gemcitabine-(C_4_-*amide*)-[anti-EGFR] with epirubicin-(C_3_-*amide*)-[anti-HER2/*neu*] formulated as a chemotherapeutic-standardized 50/50 dual-combination following a 182-hour incubation period appeared distinctly different than those detected for only epirubicin-(C_3_-*amide*)-[anti-HER2/*neu*] after a 96-hour incubation period especially at the chemotherapeutic-equivalent concentrations of 10^−10^ M, 10^−9^ M and 10^−8^ M (Figure. 4). Anti-neoplastic cytotoxicity levels for gemcitabine-(C_4_-*amide*)- [anti-EGFR] with epirubicin-(C_3_-*amide*)-[anti-HER2/*neu*] at 182-hours and epirubicin-(C_3_-*amide*)-[anti-HER2/*neu*] after a 96-hour at and between the chemotherapeutic-equivalent concentrations of 10^−14^ M and 10^−11^ M were essentially identical based on values of 5.6% and 0.4% (94.4% -vs- 99.6% residual survival) respectively (Figure. 4). Gemcitabine-(C_4_-*amide*)-[anti-EGFR] in dual-combination with epirubicin-(C_3_-*amide*)-[anti-HER2/*neu*] produced greater levels of anti-neoplastic cytotoxicity compared to epirubicin-(C_3_-*amide*)-[anti-HER2/*neu*] alone based on the measured values of 20.5% -vs-0.4% at 10^−10^ M (79.5% -vs- 99.6% residual survival); 49.5% -vs- 0.0% at 10^−9^ M (50.5% -vs- 100% residual survival); 51.3% -vs- 9.8% at 10^−8^ M (48.7% -vs- 90.2% residual survival); and 89.1% -vs-66.9% at 10^−7^ (10.9% -vs- 33.1% residual survival) respectively (Figure. 4). Both gemcitabine-(C_4_-*amide*)-[anti-EGFR] in dual-combination with epirubicin-(C_3_-*amide*)-[anti-HER2/*neu*], and epirubicin-(C_3_-*amide*)-[anti-HER2/*neu*] alone had nearly identical maximum values of 88.5% -vs- 91.9% at 10^−6^ M (11.5% -vs- 8.1% residual survival) respectively (Figure. 4). Epirubicin-(C_3_-*amide*)-[anti-HER2/*neu*] following a 182-hour incubation period produced nearly identical levels of anti-neoplastic cytotoxicity that varied between 7.7% and 9.3% residual survival for chemotherapeutic-equivalent concentrations at and between 10^−14^ M and 10^−6^ M (Figure. 4).

In the comparison of two different dual selective “targeted” delivery strategies, the combination of gemcitabine-(C_4_-*amide*)-[anti-EGFR] with epirubicin-(C_3_-*amide*)-[anti-HER2/*neu*] consistently displayed a trend for exerting greater selectively “targeted” anti-neoplastic cytotoxicity against chemotherapeutic-resistant mammary adenocarcinoma than did gemcitabine-(C_4_-*amide*)-[anti-EGFR] with gemcitabine-(C_4_-*amide*)-[anti-HER2/*neu*] at and between the chemotherapeutic-equivalent concentrations of 10^−9^ M and 10^−6^ M (Figure. 5). Relative anti-neoplastic cytotoxicity for gemcitabine-(C_4_-*amide*)-[anti-EGFR] with epirubicin-(C_3_-*amide*)-[anti-HER2/*neu*] compared to gemcitabine-(C_4_-*amide*)-[anti-EGFR] with gemcitabine-(C_4_-*amide*)-[anti-HER2/*neu*] was 49.5% -vs-24.5% (50.5% -vs- 75.5% residual survival) at 10^−9^ M; 51.3% -vs- 66.7% (48.7% -vs- 67.2% residual survival) at 10^−8^ M; 11.6% -vs- 69.9% (10.9% -vs- 30.1% residual survival) at 10^−7^ M; and 91.9% -vs- 83.7.5% (8.1% -vs- 16.3% residual survival) at 10^−6^ M respectively (Figure. 5). Essentially identical levels of selectively “targeted” anti-neoplastic cytotoxicity was measured at 10^−10^ M (79.5% -vs- 75.7% residual survival) for the dual-combinations of gemcitabine-(C_4_-*amide*)-[anti-EGFR] with epirubicin-(C_3_-*amide*)-[anti-HER2/*neu*] and gemcitabine-(C_4_-*amide*)-[anti-EGFR] with gemcitabine-(C_4_-*amide*)-[anti-HER2/*neu*] respectively (Figure. 5).

**Figure 6:**
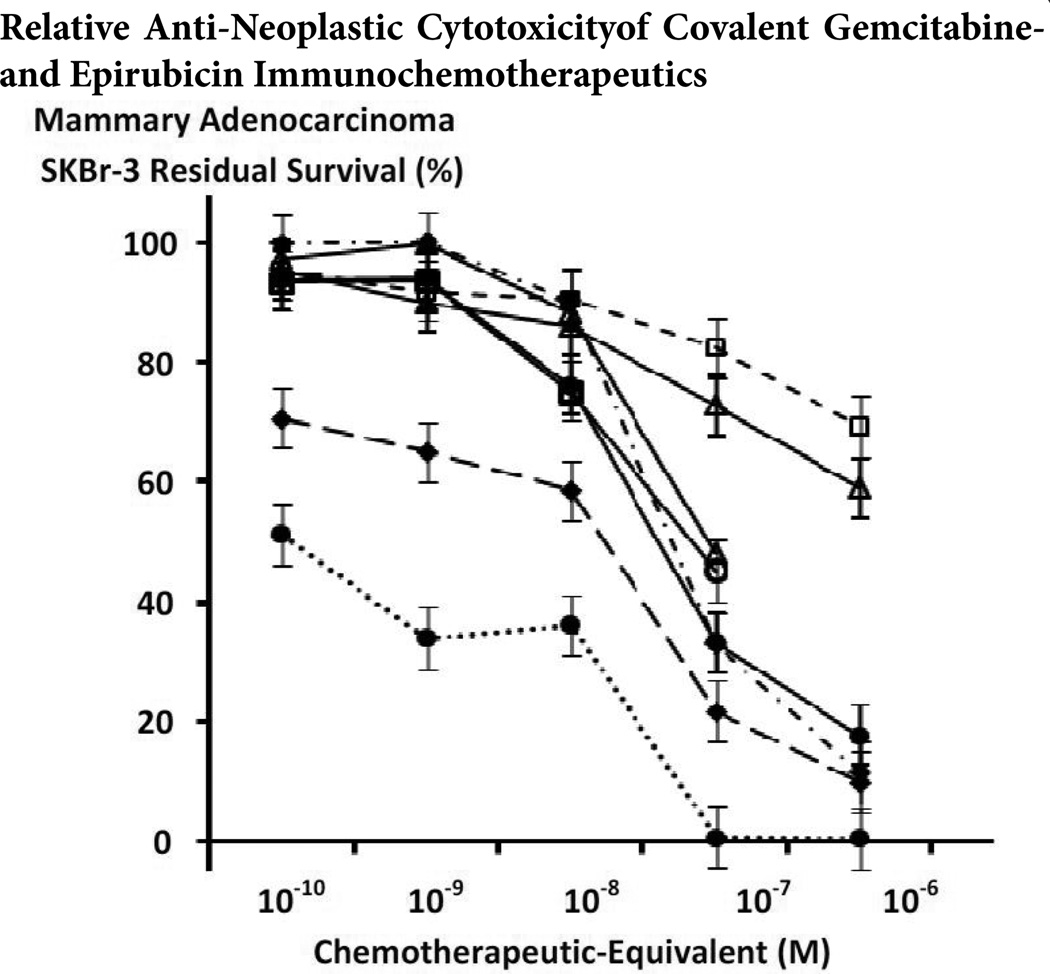
Relative anti-neoplastic cytotoxicity for gemcitabine and epirubicin against chemotherapeutic-resistant human mammary adenocarcinoma as a function of challenge duration (..●..) gemcitabine-(C4-*amide*)-[anti-EGFR] following a 182-hour incubation period;[98] (--◆--) gemcitabine-(C4-*amide*)-[anti-HER2/*neu*] following a 96-hour incubation period;[98] (--□--)* gemcitabine-(C5-carbonate)-[thiolated anti-HER2/*neu*] following a 182-hour incubation period;[97] (Δ) gemcitabine-(C4-*amide*)-[anti-HER2/*neu*] following a 96-hour incubation period;[99] (-.-◆-.-) epirubicin-(C_3_-*amide*)-[anti-HER2/*neu*] following a 96-hour incubation period;[102] (●) epirubicin-(C_3_-*amide*)-SS-[anti-HER2/*neu*] following a 96-hour incubation period;[160] (Δ)* epirubicin-(C13-imino)-[thiolated-anti-HER2/*neu*] following a 96-hour incubation period;[85] and (❍)* epirubicin-(C_3_-*amide*)-[thiolated-anti-HER2/*neu*] following a 96-hour incubation period.[66] Covalent gemcitabine or epirubicin immunochemotherapeutics were formulated at gradient chemotherapeutic-equivalent concentrations and incubated with triplicate monolayer populations of chemotherapeutic-resistant mammary adenocarcinoma (SKBr-3). Anti-neoplastic cytotoxicity was detected and measured using a MTT cell vitality assay relative to matched negative reference controls. Note: (*) = incorporation of aeromatic ring structure into synthetic bond structure of covalent immunochemotherapeutic; and (-SS-) designates incorporation of a potentially cleavable disulfide bond into the structure of covalent immunochemotherapeutic

The anti-neoplastic cytotoxicity of individual covalent epirubicin and gemcitabine immunochemotherapeutics is detectably different against chemotherapeutic resistant mammary adenocarcinoma (SKBr-3) populations (Figure. 6) [66,85,97,98,102]. Gemcitabine-(C_4_-*amide*)-[anti-EGFR] and epirubicin-(C_4_-*amide*)-[anti-HER2/*neu*] each possessed potent levels of anti-neoplastic potency as a function of both chemotherapeutic-equivalent concentration and the duration of the contact incubation period (Figures. 4, 5 and 6). Comparative evaluation reveals that gemcitabine-(C_4_-*amide*)-[anti-EGFR] was relatively more potent than many if not most analogous epirubicin[66;85;102] and especially gemcitabine[97,98] covalent immunochemotherapeutics (Figure. 6).

**Figure 7:**
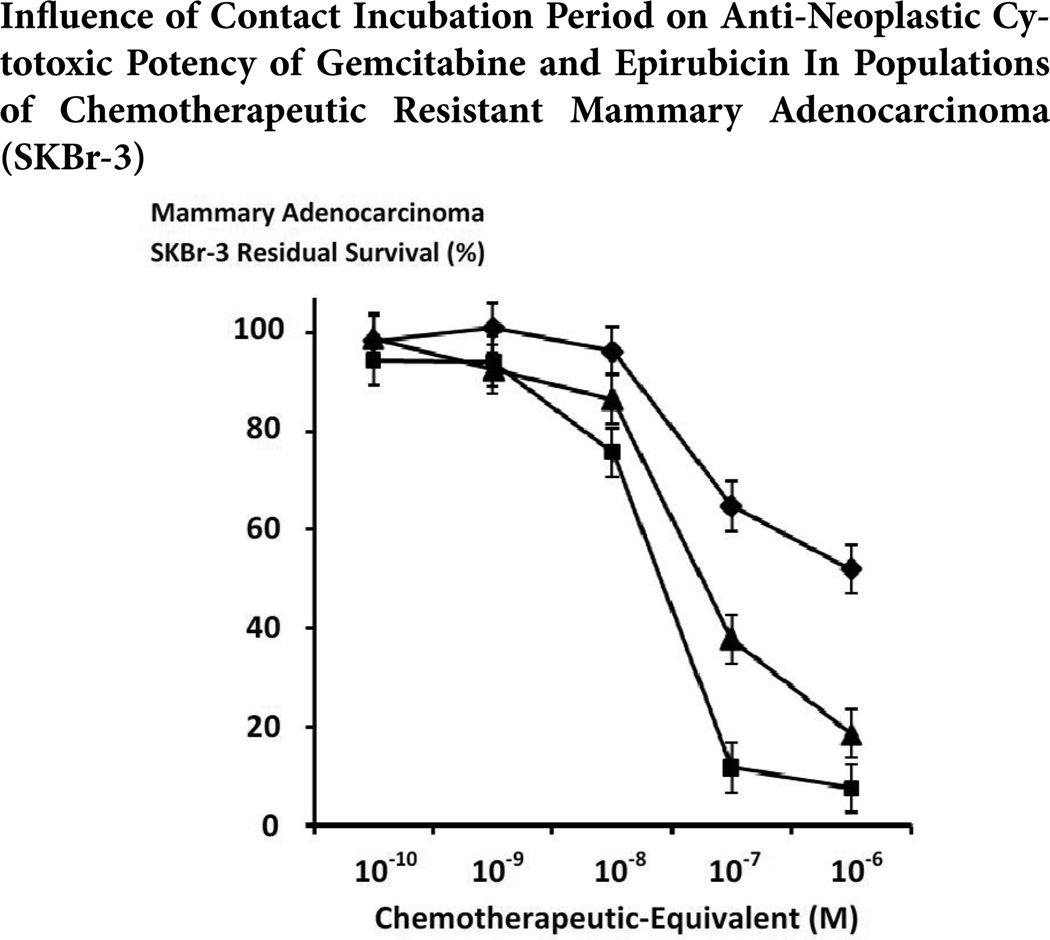
Relative anti-neoplastic cytotoxicity for gemcitabine and epirubicin against chemotherapeutic-resistant human mammary adenocarcinoma as a function of challenge duration (◆) gemcitabine following a 96-hour incubation period; (■) gemcitabine following a 182-hour incubation period; and (▲) epirubicin following a 96-hour incubation period. Gemcitabine or epirubicin formulated at gradient chemotherapeutic-equivalent concentrations were incubated in direct contact with triplicate monolayer populations of chemotherapeutic-resistant mammary adenocarcinoma (SKBr-3). Anti-neoplastic cytotoxicity was detected and measured using a MTT cell vitality assay relative to matched negative reference controls.

The relative anti-neoplastic cytotoxicity of gemcitabine against chemotherapeutic-resistant mammary adenocarcinoma (SKBr-3) following an incubation period of 96-hours was lower than levels detected following a 182-hour incubation period particularly at the chemotherapeutic-equivalent concentrations of 10^−8^ M (92.3% -vs-75.3% residual survival), 10^−7^ M (64.8% -vs- 11.7% residual survival) and 10^−6^ M (52.0% -vs-7.5% residual survival) respectively (Figure. 7). Epirubicin after a 96-hour incubation period was more potent than gemcitabine following a 96-hour incubation period which was most prominent at the chemotherapeutic-equivalent concentrations of 10^−8^ M (86.6% -vs- 92.3% residual survival), 10^−7^ M (37.9% -vs- 64.8% residual survival) and 10^−6^ M (18.5% -vs- 52.0% residual survival) respectively (Figure. 7). Gemcitabine following a 182-hour incubation period produced higher levels of anti-neoplastic cytotoxicity than epirubicin following a 96-hour incubation period which was most prominent at 10^−8^ M (75.3% -vs- 86.6% residual survival), 10^−7^ M (11.7% -vs- 37.9% residual survival) and 10^−6^ M (7.5% -vs- 18.5% residual survival) respectively (Figure. 7).

**Figure 8:**
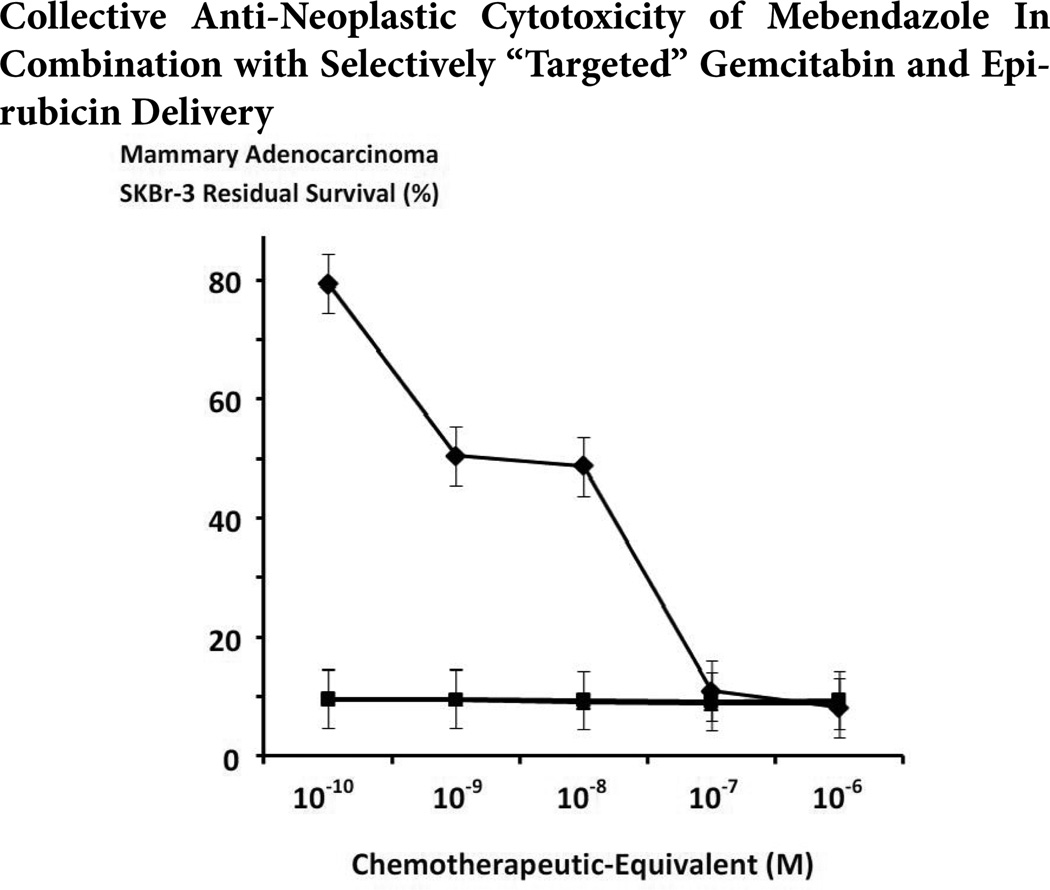
Relative anti-neoplastic cytotoxicity of gemcitabine-(C_4_-*amide*)-[anti-EGFR] in dual-combination with epirubicin-(C_3_-*amide*)-[anti-HER2/*neu*] with and without mebendazole (◆) gemcitabine-(C_4_-*amide*)-[anti-EGFR] with epirubicin-(C_3_-*amide*)-[anti-HER2/*neu*] following a 182-hour incubation period; (▲) gemcitabine-(C_4_-*amide*)-[anti-EGFR] and epirubicin-(C_3_-*amide*)-[anti-HER2/*neu*] in combination with mebendazole following a 182-hour incubation period; and (■) gemcitabine-(C_4_-*amide*)-[anti-EGFR] and epirubicin-(C_3_-*amide*)-[anti-HER2/*neu*] in combination with mebendazole following a 96-hour incubation period. Mean numerical results for (■) and (▲) are nearly identical so their marker legends are visually superimposed in the figure illustration. The covalent immunochemotherapeutic dual-combination formulated at gradient 50/50 chemotherapeutic-equivalent concentrations (+/− mebendazole 0.15µM fixed concentration) were incubated in direct contact with triplicate monolayer populations of chemotherapeutic-resist-ant mammary adenocarcinoma (SKBr-3) over a period of either 96-hours or 182-hours. Anti-neoplastic cytotoxicity was detected and measured using a MTT cell vitality assay relative to matched negative reference controls.

The anti-neoplastic cytotoxicity of gemcitabine-(C_4_-*amide*)-[anti-EGFR] with epirubicin-(C_3_-*amide*)-[anti-HER2/*neu*] formulated as a 50/50 dual-combination following a 182-hour incubation period was increased when evaluated in combination with mebendazole (0.15µM fixed final concentration) following a 96-hour incubation period (Figure. 8). The combination of mebendazole with gemcitabine-(C_4_-*amide*)-[anti-EGFR] and epirubicin-(C_3_-*amide*)-[anti-HER2/*neu*] was detectably more potent at the chemotherapeutic-equivalent concentrations of 10^−10^ M (8.7% -vs- 79.5% residual survival), 10^−9^ M (8.7% -vs- 50.5% residual survival), and 10^−8^ M (7.7% -vs- 48.7% residual survival) respectively (Figure. 8). Essentially identical levels of anti-neoplastic cytotoxicity were detected for of gemcitabine-(C_4_-*amide*)-[anti-EGFR] in dual-combination with epirubicin-(C_3_-*amide*)-[anti-HER2/*neu*] with and without mebendazole at the chemotherapeutic-equivalent concentrations of 10^−7^ M (8.1% -vs- 10.9% residual survival) and 10^−6^ M (8.6% -vs- 8.1% residual survival) respectively (Figure. 8).

**Figure 9:**
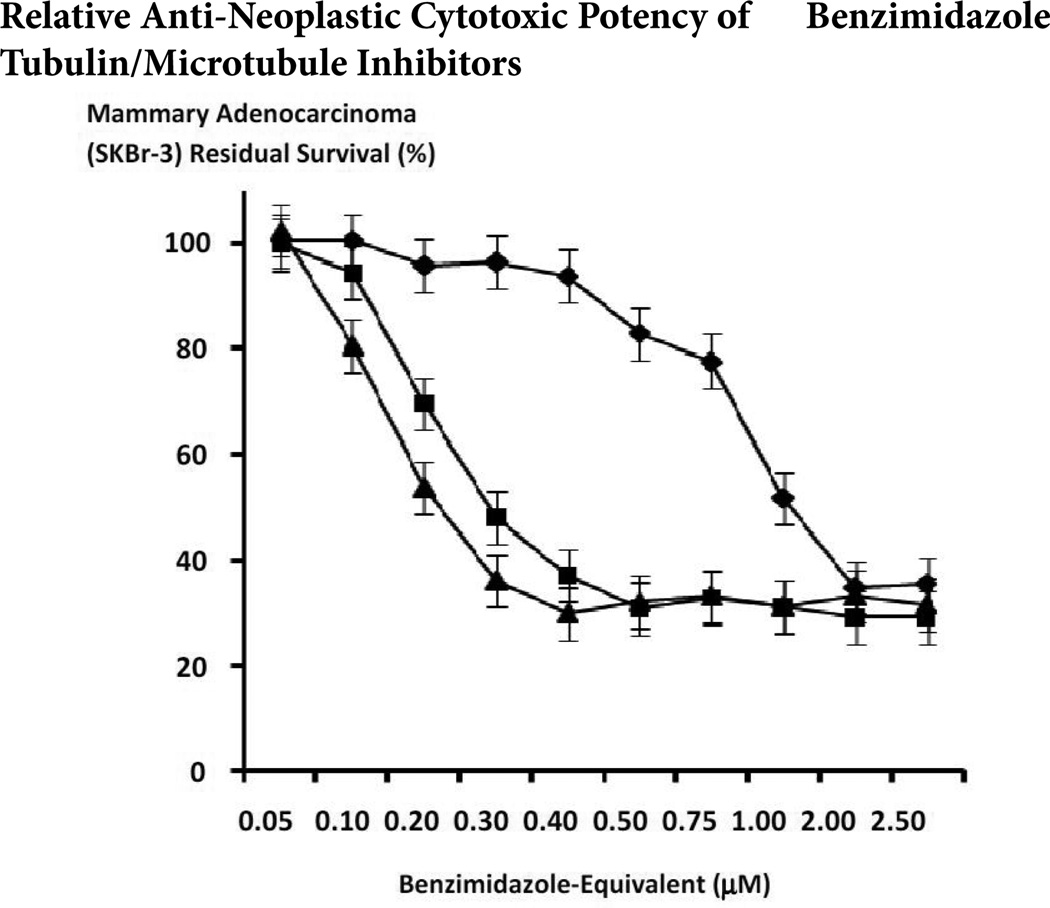
Relative anti-neoplastic cytotoxicity of benzimidazoles against chemotherapeutic-resistant human mammary adenocarcinoma (◆) albendazole; (▲) flubendazole; and (■) mebendazole. Benzimidazole tubulin/microtubule inhibitors formulated at gradient molar-equivalent concentrations were incubated in direct contact with triplicate monolayer populations of chemotherapeutic-resistant mammary adenocarcinoma (SKBr-3) over a period of 96-hours. Anti-neoplastic cytotoxicity was detected and measured using a MTT cell vitality assay relative to matched negative reference controls.

**Figure 10:**
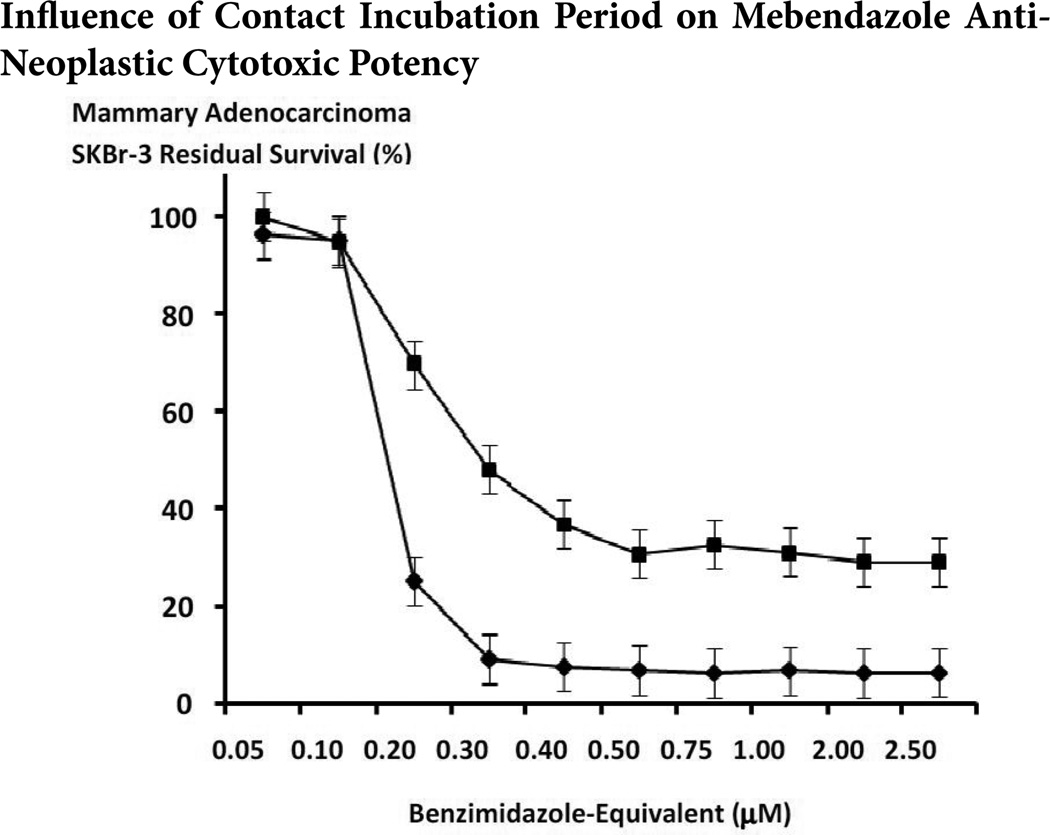
Relative anti-neoplastic cytotoxicity of mebendazole against chemotherapeutic-resistant mammary adenocarcinoma as a function of challenge duration (incubation period) (■) mebendazole following a 96-hour incubation period; and (◆) mebendazole following a 182-hour incubation period. Mebendazole formulated at gradient molar-equivalent concentrations was incubated in direct contact with triplicate monolayer populations of chemotherapeutic-resistant mammary adenocarcinoma (SKBr-3) over a period of either 96-hours or 182-hours. Anti-neoplastic cytotoxicity was detected and measured using a MTT cell vitality assay relative to matched negative reference controls.

The benzimadazole tubulin/microtubule inhibitors, albendazole, flubendazole and mebendazole exerted substantial anti-neoplastic cytotoxicity against chemotherapeutic-resistant mammary adenocarcinoma (SKBr-3) at final concentrations formulated at and between the range of 0.05µM to 2.5µM (Figure. 9). Mean anti-neoplastic cytotoxicity profiles for both flubendazole and mebendazole revealed progressive and substantial increases from approximately 0% and 0% (100% and 100% residual survival) at 0.05µM to 70.2% and 63.1% (29.8% and 36.9% residual survival) at the benzimidazole-equivalent concentration of 0.4 M respectively (Figure. 9). Mean anti-neoplastic cytotoxicity profiles for albendazole revealed a progressive increase in anti-neoplastic cytotoxicity from 6.2% (93.8% residual survival) at a benzimidazole-equivalent concentration of 0.4µM, to a near maximum of 65.4% (34.6% residual survival) at 2.0mM respectively (Figure. 9). Mean maximum cytotoxic anti-neoplastic potencies for albendazole, flubendazole and mebendazole were 64.8%, 68.7% and 70.9% (35.2%, 31.3.% and 29.1% residual survival) at the highest benzimidazole-equivalent concentration of 2.5µM (Figure. 9). Challenge of chemotherapeutic-resistant mammary adenocarcinoma (SKBr-3) with mebendazole over a longer incubation period of 182-hours compared to 96-hours resulted in substantially greater levels of anti-neoplastic cytotoxicity that were most prominent at concentrations of 0.2µM (25.2% -vs-69.6% residual survival) and 0.3µM (9.2% -vs- 48.0% residual survival) but was also evident for formulations at and between 0.4µM (7.5% -vs- 36.9% residual survival) and 2.5µM (6.4% -vs- 29.1% residual survival) respectively (Figure. 10).

## Discussion

Covalent immunochemotherapeutics can be synthesized that promote both selective “targeted” chemotherapeutic delivery, and through a variety of mechanisms exert greater levels of anti-neoplastic cytotoxicity compared to the *“free”* non-covalently bound form of the chemotherapeutic moeity. [66,85,89,92,96,102,105–107] Covalent anthracycline immunochemotherapeutics have been designed that selectively “target” chemotherapeutic delivery to, and evoke potent *ex-vivo* anti-neoplastic cytotoxicity against several different cancer cell types including mammary adenocarcinoma (anti-HER2/*neu*, anti-EGFR),[66,85] colon adenocarcinoma (anti-CEA);[93] multiple myeloma (CD38^+^, MC/CAR),[88] B-lymphoma,[87] melanoma,[89,92,96] gastric carcinoma,[108] colon carcinoma,[94] pulmonary carcinoma,[104] and other neoplastic cell types (CEA).[91,92] In direct accord with their level of *in-vitro* efficacy, similar covalent anthracycline immunochemotherapeutics reduce *in-vivo* tumor burden and prolong survival against human xenografts of gastric carcinoma,[108] breast cancer,[90] CD38 positive MC/CAR multiple myeloma,[88] B-lymphoma,[87] T-cell lymphoma,[109] colon carcinoma,[90,105,106,110] ovarian carcinoma,[105] pulmonary carcinoma,[90] metastatic melanoma,[89,96] hepatocellular carcinoma,[86] and intracerebral small-cell lung carcinoma[111–113].

The molecular design, synthetic organic chemistry reaction schemes, and anti-neoplastic cytotoxicity of gemcitabine covalently bound to large molecular weight delivery platforms has been described on a limited scale compared to analogous covalent anthracycline immunochemotherapeutics. Still fewer published investigations exist describe organic chemistry synthesis reactions for covalently bonding gemcitabine to monoclonal immunoglobulin or other fractions of biologically active protein/polypeptide[97,98]. Due to the type and relatively low number of chemical groups (sites) available within the molecular structure of gemcitabine there are only a limited number of heterobifunctional organic chemistry reaction schemes that can be been utilized to covalently bond gemcitabine to large molecular weight platforms. One potential methodology involves the creation of a covalent bond structure at the cytosine-like monoamine group of gemcitabine[80,114–117] either as a direct link to a ligand or for the purpose of creating a gemcitabine reactive intermediate. Similar molecular strategies have been employed to synthesize covalent anthracycline immunochemotherapeutics through the creation of a covalent bond at the α-monoamine (C_3_-*amino*) group of the carbohydrate moiety within the molecular composition of doxorubicin, daunorubicin, epirubicin and related anthracycline class chemotherapeutics[64,66,68,70–75,77,78,82,98,118]. Generation of a covalent bond at the C5-*methylhydroxy* group of gemcitabine represents an alternative molecular strategy for the synthesis covalent gemcitabine-ligand conjugates[97,114,117,119–123].

Gemcitabine has been covalent bonded to a number of biologically relevant ligands with binding avidity for trophic receptors like HER2/*neu* and EGFR that are frequently over-expressed by many carcinomas and adenocarcinomas including those affecting the breast. Most prominent in this regard are poly-L-glutamic acid (polypeptide configuration);[122] cardiolipin;[119,120] 1-dodecylthio-2-decyloxypropyl-3-phophatidic acid;[121,123] lipid-nucleosides;[124] N-(2-hydroxypropyl)methacryl*amide* polymer (HPMA);[80] benzodiazepine receptor ligand;[114,117] 4-(N)-valeroyl, 4-(N)-lauroyl, 4-(N)-stearoyl,[116] and anti-HER2/*neu*;[97,98] in addition to 4-fluoro[^18^F]-benzaldehyde derivative[115] for application as a diagnostic positron emitting radionuclide.

A trend was recognized for the dual-combination of gemcitabine-(C_4_-*amide*)-[anti-EGFR] with epirubicin-(C_3_-*amide*)-[anti-HER2/*neu*] to exert slightly greater levels of selectively “targeted” anti-neoplastic cytotoxicity against chemotherapeutic-resistant mammary adenocarcinoma (SKBr-3) at the chemotherapeutic-equivalent concentrations of 10^−9^ M, 10^−8^ M and 10^−7^ M compared to gemcitabine-(C_4_-*amide*)-[EGFR] alone (Figure. 4). The dual-combination of gemcitabine-(C_4_-*amide*)-[anti-EGFR] with epirubicin-(C_3_-*amide*)-[anti-HER2/*neu*] also produced greater levels of selectively “targeted” anti-neoplastic cytotoxicity compared to the dual-combination of gemcitabine-(C_4_-*amide*)-[anti-EGFR] with gemcitabine-(C_4_-*amide*)-[anti-HER2/*neu*] at the chemotherapeutic-equivalent concentrations 10^−9^ M, 10^−8^ M, 10^−7^ M and 10^−6^ M although the results at 10^−6^ M were not significantly different (Figure. 5).

A variety of molecular mechanisms and cellular processes likely account for the levels of anti-neoplastic cytotoxicity produced by dual-combinations of covalent immunochemotherapeutics. Most notable in this regard is the interdependent interactions between aspects related to cancer cell biology, and properties of the corresponding immunoglobulin component of covalent immunochemotherapeutics. Cancer cell biology variables that are influential in this regard include; [*i*] expression density of the external membrane-associated trophic receptor “targets” relative to normal tissues and organ systems; [*ii*] extent that a site on the external surface of cancer cell membranes chosen to facilitate selective “targeted” chemotherapeutic delivery ultimately undergoes internalization by mechanisms of receptor-mediated-endocytosis following the physical binding of a receptor ligand or specific immunoglobulin; [*iii*] rate at which membrane trophic receptor complexes are re-expressed and replenished following (ligand or immunoglobulin induced) internalization by mechanisms of receptor-mediated-endocytosis; and the [*vi*] degree that neoplastic cell vitality and proliferation characteristics are dependent upon over-expression of specific membrane trophic receptor complexes.

In the molecular design and organic chemistry synthesis of gemcitabine-(C_4_-*amide*)-[anti-EGFR], epirubicin-(C_3_-*amide*)-[anti-HER2/*neu*] and similar covalent immunochemotherapeutics, their corresponding immunoglobulin component can innately exert an array of properties that contribute significantly to their capacity to achieve maximum anti-neoplastic cytotoxic potency. Covalent immunochemotherapeutics like gemcitabine-(C_4_-*amide*)-[anti-EGFR][98] and epirubicin-(C_3_-*amide*)-[anti-HER2/*neu*][102] that possess binding-avidity for EGFR, HER2/*neu*, IGF-1R, VEGFR or other trophic membrane receptors uniquely or highly expressed by a neoplastic cell then their immunoglobulin component is capable of directly or indirectly suppressing neoplastic cell vitality, proliferation rate, metastatic potential, and chemotherapeutic resistance. Inhibiting the function of trophic receptors over-expressed by neoplastic cells by the immunoglobulin component of covalent immunochemotherapeutics is in part achieved through competitive inhibition of endogenous ligand binding at membrane receptor sites (e.g. (e.g. EGF ⇉| IgG::EGFR). Inhibitory effects of this type are complemented by a transient down-regulated expression, or rather a partial or complete depletion of trophic membrane receptor expression secondary to mechanisms of immunoglobulin-induced receptor-mediated-endocytosis[125].

In parallel with suppression of trophic membrane receptor function the IgG immunoglobulin component of gemcitabine-(C_4_-*amide*)-[anti-EGFR], epirubicin-(C_3_-*amide*)-[anti-HER2/*neu*], and analogous covalent immunochemotherapeutics effectively facilitates selective “targeted” chemotherapeutic delivery and continual deposition of the chemotherapeutic moiety onto the exterior surface membrane of neoplastic cell populations. The decision regarding which site or sites on the external surface membrane of neoplastic cells is to be selected to facilitate selective “targeted” chemotherapeutic delivery is important because it determines several critical attributes. In general theory and practice the immunoglobulin component of covalent immunochemotherapeutics can promote selective “targeted” chemotherapeutic delivery only if it possesses binding-avidity specifically for an antigenic “site” that is either uniquely expressed or relatively over-expressed on the external surface membrane of cancer cells compared to normal healthy tissues and organ systems. In mammary adenocarcinoma (SKBr-3) EGFR (2.2 × 10^5^/cell) is over-expressed and HER2/*neu* (1 × 10^6^/cell) is highly over-expressed compared to normal/healthy tissues and organ systems. Similar membrane-associated antigenic “sites” that are over-expressed by neoplastic cell types include CD19 (B-cell lymphoma), CD20 (chronic lymphocytic leukemia), CD22 (Non-Hodgkin lymphoma, CD30 (Hodgkin lymphoma), CD33 (acute myelogenous leukemia), CD52 (chronic lymphocytic leukemia), CD74 (multiple myeloma, B-cell lymphoma), carcinoembryonic antigen (CEA: LoVo colon carcinoma), cervical carcinoma cell-surface antigen (cervical carcinoma), chondroitin sulfate proteoglycan (metastatic melanoma), epidermal growth factor receptor (EGFR: mammary adenocarcinoma/carcinoma, metastatic melanoma, oral epidermoid carcinoma).

Due to their relatively massive size (molecular weight), gemcitabine-(C_4_-*amide*)-[anti-EGFR] and epirubicin-(C_3_-*amide*)-[anti-HER2/*neu*] are essentially incapable of passively diffusing across the intact structure of the lipid bilayer membrane in neoplastic cell populations. The immunoglobulin component of gemcitabine-(C_4_-*amide*)-[anti-EGFR], epirubicin-(C_3_-*amide*)-[anti-HER2/*neu*] and other covalent immunochemotherapeutics can facilitate not only the selective “targeted” delivery and deposition of chemotherapeutics on the external surface membrane of neoplastic cell types, but it can also initiate active trans-membrane intracellular transport of chemotherapeutic moieties. Trans-membrane intracellular transport of covalent immunochemotherapeutics is possible if their immunoglobulin component physically binds to (antigenic) “sites” on the exterior surface membrane of neoplastic cells that are known to be internalized by mechanisms similar or identical to those observed following selective binding of endogenous receptor ligands or immunoglobulin fraction (e.g. EGF or anti-EGFR → EGFR). Such qualities are often a characteristic of trophic membrane receptor complexes like EGFR, HER2/*neu*, IGF-1R, and VEGFR that are each over-expressed by several neoplastic cell types including adenocarcinomas and carcinomas that affect the breast, ovary, prostate, lung and intestine. Selective binding of endogenous receptor ligands and immunoglobulin fractions at their corresponding receptor “sites” on the external surface membrane of neoplastic cells initiates internalization by receptor-mediated-endocytosis phenomenon. [125] Given this perspective, the immunoglobulin component of covalent immunochemotherapeutics like gemcitabine-(C_4_-*amide*)-[anti-EGFR] and epirubicin-(C_3_-*amide*)-[anti-HER2/*neu*] affords several important outcomes and attributes. First, receptor-mediated-endocytosis stimulated by the binding of the immunoglobulin component reduces the risk of covalent immunochemotherapeutics like gemcitabine-(C_4_-*amide*)-[anti-EGFR] and epirubicin-(C_3_-*amide*)-[anti-HER2/*neu*] from simply coating the exterior surface membrane of neoplastic cell populations. Such a prerequisite is only necessary if the chemotherapeutic moiety exerts a mechanism-of-action that is dependent upon entry into the cytosol or nuclear environments. Second, internalization of covalent immunochemotherapeutics by receptor-mediated-endocytosis at endogenous trophic receptor “sites” aids in facilitating progressive and continual active transmembrane transport and subsequent intra-cellular accumulation of the chemotherapeutic moiety.

In concept, a uniquely high “target” expression density on the exterior surface membrane is assumed to represent a distinctly desirable characteristic for the purpose of facilitating selective “targeted” chemotherapeutic delivery because it theoretically maximizes the amount of a covalent immunochemotherapeutic deposited on the external surface membrane of a given cancer cell populations. However, from the perspective of immunoglobulin fractions with binding-avidity for trophic membrane receptors, there has been some speculation that has proposed that immunotherapeutics like anti-EGFR, anti-HER2/*neu*, anti-IGF-1R, and anti-VEGFR may be most effective when their corresponding “targets” are expressed by neoplastic cell types at intermediate instead of high or ultra-high levels. A second assumption has proposed that high “target” membrane expression densities also accelerates the rate and extent that chemotherapeutic moieties of covalent immunochemotherapeutics like gemcitabine-(C_4_-*amide*)-[anti-EGFR] and epirubicin-(C_3_-*amide*)-[anti-HER2/*neu*] are actively transported across intact external membrane structures of neoplastic cell types. Given a single trophic membrane receptor type in a single neoplastic cell type this assumption is largely considered to be relatively accurate. However, EGFR, HER2/*neu*, IGF-1R, VEGFR and other trophic membrane receptor sites in different neoplastic cell types are likely internalized by mechanisms of receptor-mediated-endocytosis at relatively unequal rates and are also subsequently re-expressed and replenished at different rates following internalization. Specific data remains rather limited about the receptor-mediated-endocytosis of covalent immunochemotherapeutics like epirubicin-[anti-HER2/*neu*][66,85,102] epirubicin-[anti-EGFR],[66] gemcitabine-[anti-HER2/*neu*],[97,98] or gemcitabine-[anti-EGFR] following their physical binding to the trophic receptors, EGFR or HER2/*neu* over-expressed by mammary adenocarcinoma (SKBr-3). However, metastatic multiple myeloma cell types are known to internally transport and subsequently metabolize approximately 8 × 10^6^ molecules of anti-CD74 monoclonal antibody per day[126]. Acknowledgement of this consideration correlates with the basic concept that selective “targeted” chemotherapeutic delivery at a single membrane-associated receptor complex and its subsequent internalization by receptor-mediated-endocytosis can result in increases in intra-cellular chemotherapeutic concentrations that approach and exceed levels 8.5×[106] to >100×[127] fold greater than those capable of being attaining by simple passive chemotherapeutic diffusion from the extracellular fluid compartment (e.g. following intravenous injection of “free” chemotherapeutic). Assumed advantages of promoting higher cytosol chemotherapeutic concentrations at least in theory is that it accelerates the rate at which neoplastic cells are resolved in-situ thereby reducing the frequency and time frame during which certain forms of chemotherapeutic-resistance can develop. In concert with these considerations, it would logically be anticipated that total overall dosage requirements would also be reduced.

Several pharmaceutical strategies exist for increasing the total amount of chemotherapeutic moiety actively transported across intact membranes of neoplastic cells and into the intracellular cytosol environment of neoplastic cells in the form of a covalent immunochemotherapeutic. Simultaneous selective “targeted” chemotherapeutic delivery of dual covalent immunochemotherapeutic combinations like gemcitabine-(C_4_-*amide*)-[anti-EGFR] or epirubicin-(C_3_-*amide*)-[anti-HER2/*neu*] that are directed at more than one receptor (antigenic) “site” expressed on the exterior surface membrane of neoplastic cell populations represents one potential approach to achieving this objective (Figures. 4 and 5). Alternatively, the expression densities of one or more membrane receptor “sites” utilized for the purpose of selectively “targeted” chemotherapeutic delivery can be enhanced by up-regulating their translation[128;129]. Complementary cancer cell biology based strategies include accelerating the rate at which trophic receptor “sites” are replenishment (re-expression) on the exterior surface membrane following internalization by receptor-mediated-endocytosis during the time period when neoplastic cell sub-populations remain viable. Lastly, elevating the amount of chemotherapeutic actively transported into the cytosol of individual neoplastic cells by mechanisms of receptor-mediated-endocytosis can also be increased by utilizing synthetic organic chemistry reactions and conditions that elevate the molar-incorporation index of the chemotherapeutic moiety of covalent immunochemotherapeutics.

The covalent bonding of chemotherapeutics to delivery platforms provides several somewhat passive but none the less important attributes that are directly related to molecular weight. Enhanced levels of anti-neoplastic cytotoxicity for gemcitabine-(C_4_-*amide*)-[anti-EGFR] or epirubicin-(C_3_-*amide*)-[anti-HER2/*neu*] against chemotherapeutic-resistant mammary adenocarcinoma (SKBr-3) and potentially other chemotherapeutic-resistant cancer populations can be attributed to covalent bonding of the chemotherapeutic moieties to a delivery platform that has a much larger molecular weight (e.g. IgG MW = 150,000 Da -vs- gemcitabine MW = 263.198 Da). Covalent bonding of chemotherapeutics to large molecular weight platforms of this size imparts physical alterations that through mechanisms of steric hinderance inhibit the biological function of entities that can utilize the “free” form of chemotherapeutics as a substrate. In this manner, the biochemical activity of degradative enzymes is suppressed (e.g. gemcitabine inactivation by cytosine deaminase) as is the capacity of P-glycoprotein (MDR-1: multi-drug resistance protein)[121] to extracellular chemotherapeutic transport when it functions as a non-selective trans-membrane efflux “pump” complex (commonly associated with mechanisms of chemotherapeutic-resistance)[130–135]. Such a phenomenon may in part reflect the observation that 24-hours post selective “targeted” delivery, a relatively large proportion of an anthracycline (>50%) is retained intracellularly[106] where it becomes primarily associated with membrane structures or it can be found distributed throughout the cytosol environment[125,136]. In this context, “free” non-conjugated anthracycline following passive diffusion across intact lipid bi-layer membranes is primarily detected within complexes associated with nuclear DNA less than 30 minutes after initial exposure[125] while anthracycline liberated from covalent anthracycline immunochemotherapeutics reportedly distributes to, and accumulates within the nucleus, mitochondria and golgi compartments.[103] Acknowledgement that chemotherapeutic moieties of most, if not all covalent immunochemotherapeutics are less affected by P-glycoprotein associated extracellular transport is important from a clinical perspective because a high percentage of aggressive and resistant forms of breast cancer over-express EGFR and/or HER2/*neu*[137–139] and this characteristic is frequently associated with chemotherapeutic-resistance, elevated cancer cell survival characteristics, and increased proliferation rates (e.g. relevant to local invasiveness and metastatic dissemination) [140,141]. Resistant forms of breast cancer that over-expresses EGFR and HER2/*neu* often are less vulnerable to the cytotoxic properties of chemotherapeutics due to simultaneous over-expression of transmembrane P-glycoprotein[142–147].

Similar in concept to the large molecular weight immunoglobulin fractions decreasing the vulnerability of gemcitabine and epirubicin moieties within gemcitabine-(C_4_-*amide*)-[anti-EGFR] or epirubicin-(C_3_-*amide*)-[anti-HER2/*neu*] to the influence of P-glycorpotein, this same attributes imposes steric hinderance phenomenon that suppresses chemotherapeutic metabolism. As a consequence, chemotherapeutic moieties like gemcitabine that are covalently bound to immunoglobulin are less vulnerable to biochemical degradation by enzyme fractions like cytadine deaminase, and deoxycytidylate deaminase (following gemcitabine phosphorylation) which both impose rapid deamination reactions. Lastly, the large mass size of immunoglobulin fractions is greater than the glomerular filtration molecular weight cut of (MWCO = 50-kDa) which in turn effectively decreases the renal clearance (rate and extent) of chemotherapeutic moieties associated with covalent immunochemotherapeutics in a manner that substantially prolongs their plasma pharmacokinetic profile.

The covalent immunochemotherapeutics, gemcitabine-(C_4_-*amide*)-[anti-HER2/*neu*],[98] and epirubicin-(C_3_-*amide*)-[anti-HER2/*neu*][102] both individually or in dual-combination with one another can potentially evoke additive and synergistic planes of anti-neoplastic cytotoxicity.

### Level-1

Additive or synergistic levels of selectively “targeted” anti-neoplastic cytotoxicity can be attained when two different chemotherapeutic moieties are utilized in the organic chemistry synthesis of two different covalent immunochemotherapeutics. Dual selective “targeted” delivery of gemcitabine and epirubicin at two different trophic membrane receptors over-expressed (EGFR) or highly over-expressed (HER2/*neu*) by chemotherapeutic-resistant mammary adenocarcinoma (SKBr-3) collectively serve as a prototype strategy for attaining similar properties through the simultaneous application of gemcitabine-(C_4_-*amide*)-[anti-EGFR] with epirubicin-(C_3_-*amide*)-[anti-HER2/*neu*] based on the levels of anti-neoplastic cytotoxicity detected at and between the chemotherapeutic equivalent concentrations of 10^−9^ M and 10^−7^ M (Figure. 4). In this example, additive and synergistic properties of anti-neoplastic cytotoxicity are dependent upon the collective innate mechanisms-of-action associated with the dual gemcitabine and epirubicin combination of chemotherapeutics; their simultaneous or synchronized internalization by mechanisms of receptor-mediated-endocytosis; and the unique biological characteristics of chemotherapeutic-resistant mammary adenocarcinoma (SKBr-3). The anthracyclines in general are a highly potent class of chemotherapeutic that have been co-administered with gemcitabine for the therapeutic management of several different forms of advanced neoplastic disease[148] including breast cancer,[149] renal carcinoma,[150] and leiomyosarcoma[151]. Gemcitabine exerts synergistic anti-neoplastic cytotoxicity when applied in combination with a number of conventional “small molecular weight” chemotherapeutics including oxaliplatin,[152] 5-fluorouracil (5-FU),[153] pemetrexed,[154,155] hydroxyurea,[156,157] bortezomib,[158] and sorafenib[159].

General pharmacology guidelines advocate that different chemotherapeutics should be utilized in dual-combinations that ideally possess mechanisms-of-action that are complementary in effect and distinctly different in order to avoid competitive inhibition phenomenon. However, chemotherapeutic moieties covalently bound to large molecular weight platforms that facilitate selective “targeted” delivery are usually associated with a decreased frequency and severity of sequelae that are commonly induced by the “free” non-protein bound form of the chemotherapeutic agent. Because covalent immunochemotherapeutics impose a lower frequency and severity of sequelae it then becomes possible to utilized combinations of two or more chemotherapeutic moieties that normally could not previously be administered together in a “free” non-protein bound form due to dose-limiting sequelae. In effect, covalently bonding multiple different chemotherapeutics to large molecular weight delivery platforms therefore functions as a strategy for broadening the therapeutic spectrum of different chemotherapeutic combinations (e.g. n = ≥2) because of their wider margin-of-safety. Given this perspective covalently bonding chemotherapeutics to large molecular weight delivery platforms provides a potential opportunity to also administer total chemotherapeutic dosage levels for the resolution of resistant forms of neoplastic disease that could not be safely resolved with the same total dosage of the “free” non-protein bound form of the chemotherapeutic agent.

### Level-2

Simultaneous or staggered inhibition of EGFR (over-expressed) and HER2/*neu* (highly over-expressed) function on the external surface membrane of mammary adenocarcinoma (SKBr-3) utilizing multiple covalent immunochemotherapeutics represents an approach for attaining additive and synergistic levels of anti-neoplastic efficacy. Selectively “targeted” inhibition of different membrane trophic receptors with multiple covalent immunochemotherapeutics like the dual-combination of gemcitabine-(C_4_-*amide*)-[anti-EGFR] with epirubicin-C_3_-*amide*)-[anti-HER2/*neu*] is important because EGFR, HER2/*neu*, IGFR, and VEGFR are over-expressed by many neoplastic cell types where they directly or indirectly regulate proliferation rate, metastatic characteristics, and chemotherapeutic resistance. Inhibiting the function of more than one trophic receptor over-expressed on the exterior surface membrane of neoplastic cell populations is also important because a single immunoglobulin fraction like anti-EGFR, anti-HER2/*neu*, anti-IGFR, and anti-VEGFR usually are only capable of reducing cancer cell vitality and decreasing rates of proliferation but are generally incapable of evoking cytotoxic resolution of neoplastic disease[15,16,30–34].

The opportunity to achieve synergistic levels of anti-neoplastic efficacy is at least theoretically greatest when two or more trophic receptors utilized to selectively “target” chemotherapeutic delivery has a distinctly different effect on neoplastic cell biology. Alternatively it is assumed that inhibiting the function of two different trophic receptors that each have essentially an identical influence or effect on cancer cell biology would probably not produce a synergistic effect but could promote greater suppression of neoplastic cell vitality and proliferation than is possible with just a single anti-trophic receptor immunoglobulin. Although the *in-vivo* immune-mediated anti-neoplastic properties of anti-trophic receptor immunoglobulins is highly relevant, such processes are unfortunately challenging to collectively simulate and difficult accurately detect during the relatively brief incubation periods employed for evaluating the *ex-vivo* potency of many if not most covalent immunochemotherapeutics[66,85,97–99,102,160].

### Level-3

Additive and synergistic levels of anti-neoplastic cytotoxicity can be attained when immunoglobulin fractions like anti-EGFR, anti-HER2/*neu*, anti-IGFR, and anti-VGFR are selected for the organic chemistry synthesis of covalent immunochemotherapeutics possesses because of their innate anti-neoplastic properties. Multiple levels of additive and synergistic anti-neoplastic cytotoxicity can ultimately be attained when two different covalent immunochemotherapeutics are applied in dual-combination within one another when they are composed of different chemotherapeutic moieties, and are selectively “targeted” for delivery at different membrane trophic receptor types. In instances where Simultaneous selective “targeted” chemotherapeutic delivery (e.g. gemcitabine and epirubicin) in dual-combination with inhibition of trophic-receptor function especially when they are over-expressed (e.g. SKBr-3: EGFR) or highly over-expressed (e.g. SKBr-3: HER2/*neu*) represents an opportunity for generating additive or synergistic levels of anti-neoplastic cytotoxicity[45–49,52–55,58,59,161,162]. Additive or synergistic interactions of this type have been detected between anti-HER2/*neu* when applied in simultaneous combination with cyclophosph*amide*,[46,48] docetaxel,[48] doxorubicin,[46;48] etoposide,[48] methotrexate,[48] paclitaxel,[46;48] or vinblastine[48]. Similar to anti-HER2/*neu*,[46,48–52] other trophic receptor site inhibitors including anti-EGFR,[53–55] anti-IGFR-1,[56,57] and anti-VEGFR[45,58,59] also create additive and synergistic levels of anti-neoplastic cytotoxicity when applied in combination with conventional chemotherapeutic agents. In the dual-combination of gemcitabine-(C_4_-*amide*)-[anti-EGFR] with epirubicin-(C_3_-*amide*)-[anti-HER2/*neu*] the possible levels of additive or synergistic interactions between chemotherapeutic moieties and immunoglobulin fractions would include; [*i*] gemcitabine and anti-EGFR, [*ii*] gemcitabine and anti-HER2/*neu*; [*iii*] gemcitabine, anti-EGFR, and anti-HER2/*neu*; [*iv*] epirubicin and anti-EGFR, [*v*] epirubicin and anti-HER2/*neu*; [*vi*] epirubicin, anti-EGFR, and anti-HER2/*neu*; [*vii*] gemcitabine, epirubicin and anti-EGFR; [*viii*] gemcitabine, epirubicin and anti-HER2/*neu*; and/or [*ix*] gemcitabine, epirubicin, anti-EGFR and anti-HER2/*neu*.

### Level-4

Simultaneous *in-vivo* selective “targeted” delivery of chemotherapeutic at trophic receptor sites (highly) over-expressed on the exterior surface membrane of neoplastic cell populations utilizing covalent immunochemotherapeutics provides affords attaining additional planes of additive and synergistic anti-neoplastic cytotoxicity. When dual-combinations of covalent immunochemotherapeutics like gemcitabine-(C_4_-*amide*)-[anti-EGFR] and epirubicin-(C_3_-*amide*)-[anti-HER2/*neu*] are selectively “targeted” *in-vivo* at EGFR and HER2/*neu* that are highly over-expressed by chemotherapeutic-resistant mammary adenocarcinoma (SKBr-3) it results in initiation of several innate immune responses that produce a variable degree of anti-neoplastic cytotoxicity. Most prominent in this regard is an additive or synergistic levels of anti-neoplastic cytotoxicity potentially attained through the combined interdependent effects of several immune-dependent processes that can include; [*i*] complement-mediated cytolysis; [*ii*] opsonization subsequent to the formation of IgG/receptor/complement complexes on the exterior surface of neoplastic cell membranes (e.g. macrophage mediated phagocytosis); and [*iii*] antibody dependent cell-mediated cytotoxicity (ADCC) which is classically mediated through natural killer lymphocytes (NK cells) but participation in this response can also include macrophages, *neu*trophils and eosinophils. In ADCC responses the immune cells involved in this phenomenon release cytotoxic components that are known to additively and synergistically enhance the cytotoxic anti-neoplastic activity of conventional chemotherapeutic agents.[163] Recognition of the collective role that different immune-dependent responses have in contributing to additive and synergistic levels of anti-neoplastic potency at least in part delineates how covalent immunochemotherapeutics frequently evoke greater efficacy when implemented *in-vivo* compared to *ex-vivo* tissue culture based models for neoplastic disease even when the same identical cancer cell type (xenographs) are utilized[108,164,165].

Due to potential for complement-mediated lysis, ADCC and opsonization to all contribute to enhance the levels of anti-neoplastic cytotoxicity of covalent immunochemotherapeutics like gemcitabine-(C_4_-*amide*)-[anti-EGFR] and epirubicin-(C_3_-*amide*)-[anti-HER2/*neu*], it is technically very difficult to simultaneously and accurately simulate these three immune-dependent responses utilizing *ex-vivo* models for neoplastic disease. In clinical environments immunoglobulin fractions when utilized for selective “targeted” delivery of therapeutic pharmaceuticals or diagnostic imaging agents in nuclear medicine frequently are biochemically modified with enzymes like papain in order to cleave (remove) the Fc segment of the IgG molecule. In effect, such biochemical modifications minimize non-selective binding at Fc receptors expressed by cells within the RE system (mononuclear phagocytic system) physically residing within the spleen and liver. Unfortunately, such biochemical modifications create a covalent immunochemotherapeutic composed of just F(ab’)_2_ or Fab’ fragment which would have less of a capacity to induce activation of the complement cascade (e.g. C9 cytolysis, C3b/C4b opsonization), neoplastic cell opsonization (e.g. macrophage Fc receptor dependent binding), or ADCC phenomenon (e.g. NK lymphocyte Fc receptor dependent binding).

### Level-5

Dual-combinations of gemcitabine-(C_4_-*amide*)-[anti-EGFR] and epirubicin-(C_3_-*amide*)-[anti-HER2/*neu*] *in-vivo* presents an opportunity to potentially attain still another plane of additive and synergistic anti-neoplastic cytotoxicity that involves; [*i*] gemcitabine, trophic membrane receptor inhibition, and innate immune response activation; and/or [*ii*] epirubicin, trophic membrane receptor inhibition, and innate immune response activation. In support of this concept, immune cell populations that are involved in ADCC phenomenon release cytotoxic components that are known to additively and synergistically enhance the cytotoxic anti-neoplastic activity of conventional chemotherapeutic agents[163]. Undoubtedly, other immune responses also contribute to the anti-neoplastic properties of many conventional chemotherapeutic agents. Recognition of the phenomenon where different immune-dependent responses become a significant component of additive and synergistic anti-neoplastic cytotoxicity phenomenon in active partnership with chemotherapeutics and trophic receptor inhibition at least in part delineates how covalent immunochemotherapeutics frequently evoke greater efficacy when implemented *in-vivo* compared to their evaluation in *ex-vivo* tissue culture based models for neoplastic disease even when the same identical cancer cell type (xenographs) are utilized[108,164,165]. Each of the qualities and properties discussed for selective “targeted” chemotherapeutic delivery and additive or synergistic interactions that can be evoked by gemcitabine-(C_4_-*amide*)-[anti-EGFR] and epirubicin-(C_3_-*amide*)-[anti-HER2/*neu*] collectively serve to explain how the dual-combination of these two covalent immunochemotherapeutics produced additive levels of anti-neoplastic cytotoxicity against chemotherapeutic-resistant mammary-adenocarcinoma (SKBr-3) when utilized as an *ex-vivo* model for neoplastic disease (Figures. 4, 5 and 7). Basis for this conclusion is based on the observation that when gemcitabine-(C_4_-*amide*)-[anti-EGFR] and epirubicin-(C_3_-*amide*)-[anti-HER2/*neu*] were formulated as a 50:50 chemotherapeutic-equivalent combination the anti-neoplastic cytotoxicity levels were intermediate between levels detected for each of the two individual covalent immunochemotherapeutics (Figure. 4).

Each of the qualities and properties of selective “targeted” chemotherapeutic delivery and complementary interactions afforded by gemcitabine-(C_4_-*amide*)-[anti-EGFR] and epirubicin-(C_3_-*amide*)-[anti-HER2/*neu*] collectively serve to explain how the dual-combination of these two covalent immunochemotherapeutics have the potential to induced levels of anti-neoplastic cytotoxicity that were greater than for either of the covalent immunochemotherapeutics alone (Figure. 4). Such a consideration is particularly relevant in scenarios where neoplastic cell populations are in direct contact with gemcitabine-(C_4_-*amide*)-[anti-EGFR] and epirubicin-(C_3_-*amide*)-[anti-HER2/*neu*] over a prolonged period of time and especially following *in-vivo* administration (e.g. IV injection).

Several variables could be have been modified to increase and maximize the anti-neoplastic cytotoxicity of gemcitabine-(C_4_-*amide*)-[anti-EGFR] in dual-combination with epirubicin-(C_3_-*amide*)-[anti-HER2/*neu*].

- Almost invariably, levels of anti-neoplastic cytotoxicity can be increased by prolonging the *ex-vivo* incubation period during which time neoplastic cells are in direct and simultaneous contact with each individual covalent immunochemotherapeutic.
- A different human neoplastic cell type could have been applied to access anti-neoplastic cytotoxicity of gemcitabine-(C_4_-*amide*)-[anti-EGFR] in dual-combination with epirubicin-(C_3_-*amide*)-[anti-HER2/*neu*]. In contrast to chemotherapeutic-resistant mammary adenocarcinoma (SKBr-3) anti-neoplastic cytotoxicity of gemcitabine-(C_4_-*amide*)-[anti-EGFR] in dual-combination with epirubicin-(C_3_-*amide*)-[anti-HER2/*neu*] would likely have been higher if it had been measured utilizing an entirely different neoplastic cell type such as pancreatic carcinoma,[166] small-cell lung carcinoma,[167] neuroblastoma,[168] or leukemia/lymphoid[123;169] populations because of their relatively higher gemcitabine sensitivity. Similarly, human promyelocytic leukemia,[121;123] T-4 lymphoblastoid clones,[123] glioblastoma;[121;123] cervical epitheliod carcinoma,[123] colon adenocarcinoma,[123] pancreatic adenocarcinoma,[123] pulmonary adenocarcinoma,[123] oral squamous cell carcinoma,[123] and prostatic carcinoma[80] have been found to be sensitive to gemcitabine and gemcitabine-(oxyether phopholipid) covalent chemotherapeutic conjugates. Within this array of neoplastic cell types, however, human mammary carcinoma (MCF-7/WT-2’)[123] and mammary adenocarcinoma (BG-1)[123] are known to be relatively more resistant to gemcitabine and gemcitabine-(oxyether phopholipid) chemotherapeutic conjugate. Presumably this pattern of diminished gemcitabine sensitivity is directly relevant to the anti-neoplastic cytotoxicity detected for gemcitabine-(C_4_-*amide*)-[anti-EGFR] in dual-combination with epirubicin-(C_3_-*amide*)-[anti-HER2/*neu*] compared to gemcitabine in chemotherapeutic-resistant mammary adenocarcinoma (SKBr-3) populations (Figure. 4).
- Analogous to the consideration that the utilization of a different neoplastic cell type could have been used that was more sensitive to epirubicin, and especially gemcitabine, a human cancer cell population could also have alternatively been selected to assess anti-neoplastic cytotoxicity of gemcitabine-(C_4_-*amide*)-[anti-EGFR] in dual-combination with epirubicin-(C_3_-*amide*)-[anti-HER2/*neu*] that was not chemotherapeutic-resistant. Majority of published descriptions to date that report in current literature the efficacy of covalent immunochemotherapeutics or analogous biopharmaceutical agents utilize human neoplastic cell populations that are chemotherapeutic-resistant. Rare exceptions have been the application of chemotherapeutic-resistant metastatic melanoma M21 (covalent daunorubicin immunochemotherapeutics synthesized using anti-chondroitin sulfate proteoglycan 9.2.27 surface marker);[89;92;170] chemotherapeutic-resistant mammary carcinoma MCF-7AdrR (covalent anthracycline-ligand chemotherapeutics utilizing epidermal growth factor (EGF) or an EDF fragment);[171] and chemotherapeutic-resistant mammary adenocarcinoma (SKBr-3) populations (epirubicin-anti-HER2/*neu*,[66,85,102] epirubicin-anti-EGFR,[66] gemcitabine-HER2/*neu*[97,98]) respectively.
- Anti-neoplastic cytotoxicity of gemcitabine-(C_4_-*amide*)-[anti-EGFR] in dual-combination with epirubicin-(C_3_-*amide*)-[anti-HER2/*neu*] would likely have been substantially greater if either cellular proliferation had been assessed with [^3^H]-thymidine, or an ATP-based assay method was alternatively applied as an analysis modality because of their reportedly >10-fold greater sensitivity in detecting early cell injury compared to MTT vitality stain based assay methods.[172,173] Despite this consideration, MTT vitality stain based assays continue to be extensively applied for the routine assessment of true anti-neoplastic cytotoxicity of chemotherapeutics covalently incorporated synthetically into molecular platforms that provide properties of selective “targeted” delivery.[66,121–123,174–179] One of the significant advantages of MTT vitality stain based assays and methods applying similar reagents is that the ability to measure lethal cytotoxic anti-anti-neoplastic activity is generally considered to be superior to the detection of early-stage cellular injury that could potentially be reversible.
- Lastly, as previously eluded to, a high degree of probability suggests that the anti-neoplastic cytotoxicity of gemcitabine-(C_4_-*amide*)-[anti-EGFR] in dual-combination with epirubicin-(C_3_-*amide*)-[anti-HER2/*neu*] would likely of been greater if their efficacy had been delineated *in-vivo* against human neoplastic xenographs in animal hosts as a model for human cancer. In such a scenario, added levels of selective “targeted” anti-neoplastic cytotoxicity is attained through antibody-dependent cell cytotoxicity (ADCC), complement mediated cytolysis, and immunoglobulin initiated opsonization similar to what has been observed with anti-CD20 and anti-CD52 administered for the treatment of certain forms of leukemia.

The benzimidazole tubulin/microtubule inhibitor, mebendazole in combination with gemcitabine-(C_4_-*amide*)-[anti-EG-FR] and epirubicin-(C_3_-*amide*)-[anti-HER2/*neu*] produced higher levels of anti-neoplastic cytotoxicity against chemotherapeutic-resistant mammary adenocarcinoma than did the dual covalent immunochemotherapeutic combination alone (Figure. 7). Such findings correlate with mebendazole additively or synergistically contributing to the anti-neoplastic cytotoxicity of epirubicin-(C_3_-*amide*)-[anti-HER2/*neu*][160] and gemcitabine-(C_4_-*amide*)-[anti-HER2/*neu*][99]. Preliminary experimental investigations have detected vulnerability of adrenocortical carcinoma (xenographs),[180] colorectal cancer,[181,182] hepatocellular carcinoma,[182,183] leukemia,[184,185] lung cancer,[186] (non-small cell[186,187]), melanoma (chemo-resistant),[188] myeloma,[185] and ovarian cancer,[183,189–191] to benzimidazole tubulin/microtubule inhibitors. The anti-neoplastic cytotoxicity of the benzimidazole class of tubulin/microtubule inhibitors against breast cancer has previously remained largely unknown. In contrast to a single report for flubendazole, the creation of mammalian chromosomal aberrations has to date not been described for either albendazole[181,189] or mebendazole[192].

The dual-combination of gemcitabine-(C_4_-*amide*)-[anti-EG-FR] with epirubicin-(C_3_-*amide*)-[anti-HER2/*neu*] presents several opportunities for inducing lower frequencies of severe *in-vivo* sequelae. The covalent bonding of chemotherapeutics to a high-molecular weight delivery platform to facilitate selective “targeted” delivery effectively avoids innocent high-level exposure of normal tissues and organ systems to the chemotherapeutic moiety thereby reducing the risk and frequency of severe dose-dependent sequelae (e.g. anthracycline cardiotoxicity). Utilization of “carrier” platforms like immunoglobulin fractions that have a significantly larger mass/size (IgG MW ≅ 150-kDa) than gemcitabin, epirubicin and other conventional small molecular weight chemotherapeutics employed in the synthesis of covalent immunochemotherapeutics also decreases the risk of chemotherapy-associated toxicity by several mechanisms. The much large molecular weight of gemcitabine-(C_4_-*amide*)-[anti-EGFR] and epirubicin-(C_3_-*amide*)-[anti-HER2/*neu*] drastically reduces the rate and extent that gemcitabine and epirubicin are removed from the plasma compartment and excreted into the urine by renal glomerular filtration (MWCO ≅ 50-kDa). In this manner highly renal-toxic chemotherapeutics can potentially be covalently bound to large molecular weight “targeting” platforms (e.g. IgG, Fab’, EGF) to increase their utility as a form of systemic anti-cancer therapy. The substantially lower rate and extent that large molecular weight covalent immunochemotherapeutics like gemcitabine-(C_4_-*amide*)-[anti-EGFR] and epirubicin-(C_3_-*amide*)-[anti-HER2/*neu*] are eliminated from the intra-vascular compartment by renal excretion in turn significantly prolongs and extends the chemotherapeutic moiety pharmacokinetic profile (plasma T_1/2_) and therefore contact with neoplastic lesions for longer periods of time per systemic dose. The potential for covalent immunochemotherapeutics to promote greater cytosol concentrations of the chemotherapeutic moiety than can be achieved by simple passive diffusion of the “free” chemotherapeutic from the extracellular fluid compartment can provide a level of safety because they provide an opportunity for more rapid resolution of neoplastic conditions at lower total dose requirements. Lastly, selectively “targeted” chemotherapeutic delivery allows the use of additive or synergistic chemotherapeutic combinations that otherwise could not previously be utilized in combination with one another due to dose-limiting sequelae.

Covalent immunochemotherapeutics like the dual-combination of gemcitabine-(C_4_-*amide*)-[anti-EGFR] with epirubicin-(C_3_-*amide*)-[anti-HER2/*neu*] at least conceptually can therefore afford the attributes of greater anti-neoplastic cytotoxicity and prolongation of chemotherapeutic moiety pharmacokinetic profiles that can subsequently serve collectively as the basis for reducing individual and total dosage requirements. Improved margins-of-safety are therefore possible with gemcitabine-(C_4_-*amide*)-[anti-EGFR], epirubicin-(C_3_-*amide*)-[anti-HER2/*neu*] and analogous covalent immunochemotherapeutics through a combination of selective “targeted” anti-neoplastic cytotoxicity that avoids high-level innocent exposure of normal tissues and organ system, and reductions in total dosage requirements necessary for disease resolution when implemented for intervention in clinical oncology. The benzimidazole tubulin/microtubule inhibitor, mebendazole can potentially complement and enhance these properties of dual gemcitabine-(C_4_-*amide*)-[anti-EGFR] with epirubicin-(C_3_-*amide*)-[anti-HER2/*neu*] combinations in part because of it’s anti-neoplastic cytotoxicity and because it may have a wider margin-of-safety than many if not most conventional chemotherapeutic agents.

## Conclusions

The covalent immunochemotherapeutics, gemcitabine-(C_4_-*amide*)-[anti-EGFR] and epirubicin-(C_3_-*amide*)-[anti-HER2/*neu*] both exerted selectively “targeted” anti-neoplastic cytotoxicity against chemotherapeutic-resistant mammary adenocarcinoma (SKBr-3) populations (Figures. 4 and 5). The simultaneous dual-combination of gemcitabine-(C_4_-*amide*)-[anti-EGFR] with epirubicin-(C_3_-*amide*)-[anti-HER2/*neu*] exerted at least additive levels of anti-neoplastic cytotoxicity which during the relatively brief incubation periods applied is primarily a result of the mechanisms-of-action for the two chemotherapeutic moieties, and also the anti-trophic properties of both anti-EGFR and anti-HER2/*neu* (Figures. 4 and 5). The biological integrity of the immunoglobulin component of the covalent gemcitabine and epirubicin immunochemotherapeutics not only directly facilitates selective “targeted” chemotherapeutic delivery, but it also initiates or induces internalization of covalent immunochemotherapeutics by mechanisms of receptor-mediated-endocytosis. Selection of an appropriate membrane-associated antigen that is known to undergo receptor-mediated-endocytosis maximizes the active transmembrane transport of chemotherapeutic moieties in covalent immunochemotherapeutics and many carcinoma and adenocarcinoma cell types highly over-express EGFR, HER2/*neu* and similar membrane associated receptor sites[125].

Simultaneous dual-combination of gemcitabine-(C_4_-*amide*)-[anti-EGFR] with epirubicin-(C_3_-*amide*)-[anti-HER2/*neu*] not only serves as a molecular strategy for increasing cytosol concentrations of small molecular weight chemotherapeutics beyond levels attainable by simple passive diffusion following intravenous infusion at clinically relevant dosages, it also represents an approach for reducing exposure of innocent tissues and organ systems. Mebendazole further complements these therapeutic attributes by evoking a higher level of anti-neoplastic cytotoxic against chemotherapeutic resistant mammary adenocarcinoma (SKBr-3) when applied in combination with both gemcitabine-(C_4_-*amide*)-[anti-EGFR] with epirubicin-(C_3_-*amide*)-[anti-HER2/*neu*] compared to the dual-combination of only the two covalent immunochemotherapeutics. Collectively, research investigations with gemcitabine-(C_4_-*amide*)-[anti-EGFR], epirubicin-(C_3_-*amide*)-[anti-HER2/*neu*] and mebendazole demonstrate therapeutic options for more effectively resolving chemotherapeutic-resistant neoplastic conditions within a more expedient time frame at lower total dosage levels.

## References

[1] Shamseddine AI, Khalifeh MJ, Mourad FH, Chehal AA, Al-Kutoubi A (2005). Comparative pharmacokinetics and metabolic pathway of gemcitabine during intravenous and intra-arterial delivery in unresectable pancreatic cancer patients. Clin Pharmacokinet.

[2] Giovannetti E, Laan AC, Vasile E, Tibaldi C, Nannizzi S (2008). Correlation between cytidine deaminase genotype and gemcitabine deaminationin blood samples. Nucleosides Nucleotides NucleicAcids.

[3] Gilbert JA, Salavaggione OE, Ji Y, Pelleymounter LL, Eckloff BW (2006). Gemcitabine pharmacogenomics: cytidine deaminase and deoxycytidylate deaminase gene resequencing and functional genomics. Clin Cancer Res.

[4] Honig A, Rieger L, Sutterlin A, Kapp M, Dietl J (2005). Brain metastases in breast cancer--an *in vitro* study to evaluate new systemic chemotherapeutic options. Anticancer Res.

[5] Balayssac D, Ferrier J, Descoeur J, Ling B, Pezet D (2011). Chemotherapy-induced peripheral neuropathies: from clinical relevance to preclinical evidence. Expert Opin Drug Saf.

[6] Ceresa C, Cavaletti G (2011). Drug transporters in chemotherapy induced peripheral neurotoxicity: current knowledge and clinical implications. Curr Med Chem.

[7] Raschi E, Vasina V, Ursino MG, Boriani G, Martoni A (2010). Anticancer drugs and cardiotoxicity: Insights and perspectives in the era of targeted therapy. Pharmacol Ther.

[8] Stavridi F, Palmieri C (2008). Efficacy and toxicity of nonpegylated liposomal doxorubicin in breast cancer. Expert Rev Anticancer Ther.

[9] Wachters FM, Van Der Graaf WT, Groen HJ (2004). Cardiotoxicity in advanced non-small cell lung cancer patients treated with platinum and non-platinum based combinations as first-line treatment. Anti cancer Res.

[10] Iarussi D, Indolfi P, Galderisi M, Bossone E (2000). Cardiac toxicity after anthracycline chemotherapy in childhood. Herz.

[11] Scully RE, Lipshultz SE (2007). Anthracycline cardiotoxicity in long-term survivors of childhood cancer. Cardiovasc Toxicol.

[12] Vantelon JM, Munck JN, Bourhis JH, Pico JL, Fadel C (2001). Trombotic microangiopathy: a new dose-limiting toxicity of high-dose sequential chemotherapy. Bone Marrow Transplant.

[13] Azad NS, Posadas EM, Kwitkowski VE, Steinberg SM, Jain L (2008). Combination targeted therapy with sorafenib and bevacizumab results in enhanced toxicity and antitumor activity. J Clin Oncol.

[14] Chang DZ, Olencki T, Budd GT, Peereboom D, Ganapathi R (2001). Phase I trial of capecitabine in combination with interferon alpha in patients with metastatic renal cancer: toxicity and pharmacokinetics. Cancer Chemother Pharmacol.

[15] Sliwkowski MX, Lofgren JA, Lewis GD, Hotaling TE, Fendly BM (1999). Nonclinical studies addressing the mechanism of action of trastuzumab (Herceptin). Semin Oncol.

[16] Lewis Phillips GD, Li G, Dugger DL, Crocker LM, Parsons KL (2008). Targeting HER2-positive breast cancer with trastuzumab-DM1, an antibody-cytotoxic drug conjugate. Cancer Res.

[17] Gong C, Yao Y, Wang Y, Liu B, Wu W (2011). Up-regulation of miR-21 mediates resistance to trastuzumab therapy for breast cancer. J Biol Chem.

[18] Scaltriti M, Eichhorn PJ, Cortés J, Prudkin L, Aura C (2011). Cyclin E amplification/over expression is a mechanism of trastuzumab resistance in HER2+ breast cancer patients. Proc Natl Acad Sci.

[19] Pandya K, Meeke K, Clementz AG, Rogowski A, Roberts J (2011). Targeting both Notch and ErbB-2 signalling pathways is required for prevention of ErbB-2-positive breast tumour recurrence. Br J Cancer.

[20] Morgillo F, Kim WY, Kim ES, Ciardiello F, Hong WK (2007). Implication of the insulin-like growth factor-IR pathway in the resistance of non-small cell lung cancer cells to treatment with gefitinib. Clin Cancer Res.

[21] Morgillo F, Woo JK, Kim ES, Hong WK, Lee HY (2006). Heterodimerization of insulin-like growth factor receptor/epidermal growth factor receptor and induction of survivin expression counteract the antitumor action of erlotinib. Cancer Res.

[22] Sartore-Bianchi A, Di Nicolantonio F, Nichelatti M, Molinari F, De Dosso S (2009). Multi-determinants analysis of molecular alterations for predicting clinical benefit to EGFR-targeted monoclonal antibodies in colorectal cancer. PLoS ONE.

[23] Weickhardt A, Tebbutt N, Mariadason J (2010). Strategies for overcoming inherent and acquired resistance to EGFR inhibitors by targeting downstream effectors in the RAS/PI3K pathway. Curr Cancer Drug.

[24] Modjtahedi H, Essapen S (2009). Epidermal growth factor receptor inhibitors in cancer treatment: advances, challenges and opportunities. Anticancer Drugs.

[25] Dempke WC, Heinemann V (2010). Ras mutational status is a biomarker for resistance to EGFR inhibitors in colorectal carcinoma. Anticancer Res.

[26] Schmitz S, Kaminsky-Forrett MC, Henry S, Zanetta S, Geoffrois L (2012). Phase II study of figitumumab in patients with recurrent and/or metastatic squamous cell carcinoma of the head and neck: clinical activity and molecular response (GORTEC 2008-02). Ann Oncol.

[27] Chi KN, Gleave ME, Fazli L, Goldenberg SL, So A (2012). A phase II pharmacodynamic study of preoperative figitumumab in patients with localized prostate cancer. Clin Cancer Res.

[28] Atzori F, Tabernero J, Cervantes A, Prudkin L, Andreu J (2011). A phase I pharmacokinetic and pharmacodynamic study of dalotuzumab (MK-0646), an anti-insulin-like growth factor-1 receptor monoclonal antibody, in patients with advanced solid tumors. Clin Cancer Res.

[29] Bitelman C, Sarfstein R, Sarig M, Attias-Geva Z, Fishman A (2013). IGF1R–directed targeted therapy enhances the cytotoxic effect of chemotherapy in endometrial cancer. Cancer Lett.

[30] Pietras RJ, Pegram MD, Finn RS, Maneval DA, Slamon DJ (1998). Remission of human breast cancer xenografts on therapy with humanized monoclonal antibody to HER-2 receptor and DNA-reactive drugs. Oncogene.

[31] Marches R, Uhr JW (2004). Enhancement of the p27Kip1-mediated antiproliferative effect of trastuzumab (Herceptin) on HER2-overexpressing tumor cells. Int J Cancer.

[32] Lin NU, Carey LA, Liu MC, Younger J, Come SE (2008). Phase II trial of lapatinib for brain metastases in patients with human epidermal growth factor receptor 2-positive breast cancer. J Clin Oncol.

[33] Cobleigh MA, Vogel CL, Tripathy D, Robert NJ, Scholl S (1999). Multinational study of the efficacy and safety of humanized anti-HER2 monoclonal antibody in women who have HER2-over-expressing metastatic breast cancer that has progressed after chemotherapy for metastatic disease. J Clin Oncol.

[34] Vogel CL, Cobleigh MA, Tripathy D, Gutheil JC, Harris LN (2002). Efficacy and safety of trastuzumab as a single agent in first-line treatment of HER2-overexpressing metastatic breast cancer. J Clin Oncol.

[35] Kute TE, Savage L, Stehle JR, Kim-Shapiro JW, Blanks MJ (2009). Breast tumor cells isolated from *in vitro* resistance to trastuzumab remain sensitive to trastuzumab anti-tumor effects *in vivo* and to ADCC killing. Cancer Immunol Immunother.

[36] Narayan M, Wilken JA, Harris LN, Baron AT, Kimbler KD (2009). Trastuzumab-induced HER reprogramming in “resistant” breast carcinoma cells. Cancer Res.

[37] Chen FL, Xia W, Spector NL (2008). Acquired resistance to small molecule ErbB2 tyrosine kinase inhibitors. Clin Cancer Res.

[38] Ritter CA, Perez-Torres M, Rinehart C, Guix M, Dugger T (2007). Human breast cancer cells selected for resistance to trastuzumab *in vivo* overexpress epidermal growth factor receptor and ErbB ligands and remain dependent on the ErbB receptor network. Clin Cancer Res.

[39] Nanda R (2007). Targeting the human epidermal growth factor receptor 2 (HER2) in the treatment of breast cancer: recent advances and future directions. Rev Recent Clin Trials.

[40] Mitra D, Brumlik MJ, Okamgba SU, Zhu Y, Duplessis TT (2009). An oncogenic isoform of HER2 associated with locally disseminated breast cancer and trastuzumab resistance. Mol Cancer Ther.

[41] Köninki K, Barok M, Tanner M, Staff S, Pitkänen J (2010). Multiple molecular mechanisms underlying trastuzumab and lapatinib resistance in JIMT-1 breast cancer cells. Cancer Lett.

[42] Oliveras-Ferraros C, Vazquez-Martin A, Cufí S, Torres-Garcia VZ, Sauri-Nadal T (2011). Inhibitor of Apoptosis (IAP) survivin is indispensable for survival of HER2 gene-amplified breast cancer cells with primary resistance to HER1/2-targeted therapies. Biochem Biophys Res Commun.

[43] Barok M, Tanner M, Köninki K, Isola J (2011). Trastuzumab-DM1 causes tumour growth inhibition by mitotic catastrophe in trastuzumab-resistant breast cancer cells *in vivo*. Breast Cancer Research.

[44] Oliveras-Ferraros C, Vazquez-Martin A, Martin-Castilló B, Pérez-Martínez MC, Cufí S (2010). Pathway-focused proteomic signatures in HER2-overexpressing breast cancer with a basal-like phenotype: new insights into de novo resistance to trastuzumab (Herceptin). Int J Oncol.

[45] García-Sáenz JA, Martín M, Calles A, Bueno C, Rodríguez L (2008). Bevacizumab in combination with metronomic chemotherapy in patients with anthracycline- and taxane-refractory breast cancer. J Chemother.

[46] Slamon DJ, Leyland-Jones B, Shak S, Fuchs H, Paton V (2001). Use of chemotherapy plus a monoclonal antibody against HER2 for metastatic breast cancer that overexpresses HER2. N Engl J Med.

[47] Harris CA, Ward RL, Dobbins TA, Drew AK, Pearson S (2011). The efficacy of HER2-targeted agents in metastatic breast cancer: a meta-analysis. Ann Oncol.

[48] Pegram MD, Lopez A, Konecny G, Slamon DJ (2000). Trastuzumab and chemotherapeutics: drug interactions and synergies. Semin Oncol.

[49] Slamon D, Pegram M (2001). Rationale for trastuzumab (Herceptin) in adjuvant breast cancer trials. Semin Oncol.

[50] Boone JJ, Bhosle J, Tilby MJ, Hartley JA, Hochhauser D (2009). Involvement of the HER2 pathway in repair of DNA damage produced by chemotherapeutic agents. Mol Cancer Ther.

[51] Meden H, Beneke A, Hesse T, Novophashenny I, Wischnewsky M (2001). Weekly intravenous recombinant humanized anti-P185HER2 monoclonal antibody (herceptin) plus docetaxel in patients with metastatic breast cancer: a pilot study. Anticancer Res.

[52] Winer EP, Burstein HJ (2001). New combinations with Herceptin in metastatic breast cancer. Oncology.

[53] Kim S, Prichard CN, Younes MN, Yazici YD, Jasser SA (2006). Cetuximab and irinotecan interact synergistically to inhibit the growth of orthotopic anaplastic thyroid carcinoma xenografts in nude mice. Clin Cancer Res.

[54] Landriscina M, Maddalena F, Fabiano A, Piscazzi A, La Macchia O (2010). Erlotinib enhances the proapoptotic activity of cytotoxic agents and synergizes with paclitaxel in poorly-differentiated thyroid carcinoma cells. Anticancer Res.

[55] Ciardiello F, Bianco R, Damiano V, De Lorenzo S, Pepe S (1999). Antitumor activity of sequential treatment with topotecan and anti-epidermal growth factor receptor monoclonal antibody C225. Clin Cancer Res.

[56] Quek R, Wang Q, Morgan JA, Shapiro GI, Butrynski JE (2011). Combination mTOR and IGF-1R inhibition: phase I trial of everolimus and figitumumab in patients with advanced sarcomas and other solid tumors. Clin Cancer Res.

[57] Stacchiotti S, Negri T, Palassini E, Conca E, Gronchi A (2010). Sunitinib malate and figitumumab in solitary fibrous tumor: patterns and molecular bases of tumor response. Mol Cancer Thhydrazone derivativeser.

[58] Lynn KD, Udugamasooriya DG, Roland CL, Castrillon DH, Kodadek TJ (2010). GU81, a VEGFR2 antagonist peptoid, enhances the anti-tumor activity of doxorubicin in the murine MMTV-PyMT transgenic model of breast cancer. BMC Cancer.

[59] Zhang L, Yu D, Hicklin DJ, Hannay JA, Ellis LM (2002). Combined anti-fetal liver kinase 1 monoclonal antibody and continuous low-dose doxorubicin inhibits angiogenesis and growth of human soft tissue sarcoma xenografts by induction of endothelial cell apoptosis. Cancer Res.

[60] Kaneko T, Willner D, Monkovíc I, Knipe JO, Braslawsky GR (1991). New hydrazone derivatives of adriamycin and their immunoconjugates--a correlation between acid stability and cytotoxicity. Bioconjugate Chem.

[61] Di Stefano G, Lanza M, Kratz F, Merina L, Fiume L (2004). A novel method for coupling doxorubicin to lactosaminated human albumin by an acid sensitive hydrazone bond: synthesis, characterization and preliminary biological properties of the conjugate. Eur J Pharm Sci.

[62] Kratz F, Warnecke A, Scheuermann K, Stockmar C, Schwab J (2002). Probing the cysteine-34 position of endogenous serum albumin with thiol-binding doxorubicin derivatives. Improved efficacy of an acid-sensitive doxorubicin derivative with specific albumin-binding properties compared to that of the parent compound. J Med Chem.

[63] Unger C, Häring B, Medinger M, Drevs J, Steinbild S (2007). Phase I and pharmacokinetic study of the (6-maleimidocaproyl) hydrazone derivative of doxorubicin. Clin Cancer Res.

[64] Mazuel C, Grove J, Gerin G, Keenan KP (2003). HPLC-MS/MS determination of a peptide conjugate prodrug of doxorubicin, and its active metabolites, leucine-doxorubicin and doxorubicin, in dog and rat plasma. J Pharm Biomed Anal.

[65] Greenfield RS, Kaneko T, Daues A, Edson MA, Fitzgerald KA (1990). Evaluation *in vitro* of adriamycin immunoconjugates synthesized using an acid-sensitive hydrazone linker. Cancer Res.

[66] Coyne CP, Ross MK, Bailey JG (2009). Dual potency anti-HER2/*neu* and anti-EGFR anthracycline immunoconjugates in chemotherapeutic-resistant mammary carcinoma combined with cyclosporin A and verapamil P-glycoprotein inhibition. J Drug Targeting.

[67] Lau A, Bérubé G, Ford CH, Gallant M (1995). Novel doxorubicin-monoclonal anti-carcinoembryonic antigen antibody immunoconjugate activity *in vitro*. Bioorganic and Medicinal Chemistry.

[68] Kruger M, Beyer U, Schumacher P, Unger C, Zahn H (1997). Synthesis and Stability of Four Maleimide Derivatives of the Anticancer Drug Doxorubicin for the Preparation of Chemoimmunoconjugates. Chem Pharm Bull.

[69] Furgeson DY, Dreher MR, Chilkoti A (2006). Structural optimization of a “smart” doxorubicin-polypeptide conjugate for thermally targeted delivery to solid tumors. J Control Release.

[70] Liang JF, Yang VC (2005). Synthesis of doxorubicin-peptide conjugate with multidrug resistant tumor cell killing activity. Bioorganic and Medicinal Chemistry Letters.

[71] Sirova M, Strohalm J, Subr V, Plocova D, Rossmann P (2007). Treatment with HPMA copolymer-based doxorubicin conjugate containing human immunoglobulin induces long-lasting systemic anti-tumour immunity in mice. Cancer Immunol Immunother.

[72] Wong BK, DeFeo-Jones D, Jones RE, Garsky VM, Feng DM (2001). PSA-specific and non-PSA-specific conversion of a PSA-targeted peptide conjugate of doxorubicin to its active metabolites. Drug Metab Dispos.

[73] Bidwell GL, Davis AN, Fokt I, Priebe W, Raucher D (2007). A thermally targeted elastin-like polypeptide-doxorubicin conjugate overcomes drug resistance. Invest New Drugs.

[74] Abu Ajaj K, Graeser R, Fichtner I, Kratz F (2009). *In vitro* and *in vivo* study of an albumin-binding prodrug of doxorubicin that is cleaved by cathepsin B. Cancer Chemother Pharmacol.

[75] Ryppa C, Mann-Steinberg H, Fichtner I, Weber H, Satchi-Fainaro R (2008). *In vitro* and *in vivo* evaluation of doxorubicin conjugates with the divalent peptide E-[c(RGDfK)2] that targets integrin alphavbeta3. Bioconjugate Chem.

[76] Huang YF, Shangguan D, Liu H, Phillips JA, Zhang X (2009). Molecular assembly of an aptamer-drug conjugate for targeted drug delivery to tumor cells. Chem Bio Chem.

[77] Ren Y, Wei D, Zhan X (2005). Inhibition of P-glycoprotein and increasing of drug-sensitivity of a human carcinoma cell line (KB-A-1) by an antisense oligodeoxynucleotide-doxorubicin conjugate *in vitro*. Biotechnol Appl Biochem.

[78] Ren Y, Zhan X, Wei D, Liu J (2004). *In vitro* reversal MDR of human carcinoma cell line by an antisense oligodeoxynucleotide-doxorubicin conjugate. Biomed Pharmacother.

[79] Kovar L, Etrych T, Kabesova M, Subr V, Vetvicka D (2010). Doxorubicin attached to HPMA copolymer via *amide* bond modifies the glycosylation pattern of EL4 cells. Tumour Biol.

[80] Lammers T, Subr V, Ulbrich K, Peschke P, Huber PE (2009). Simultaneous delivery of doxorubicin and gemcitabine to tumors *in vivo* using prototypic polymeric drug carriers. Biomaterials.

[81] Krakovicova H, Ethch T, Ulbrich K (2009). HPMA-based polymer conjugates with drug combinations. Eur J Pharmacol.

[82] Cao N, Feng SS (2008). Doxorubicin conjugated to D-alphatocopheryl polyethylene glycol 1000 succinate (TPGS): conjugation chemistry, characterization, *in vitro* and *in vivo* evaluation. Biomaterials.

[83] Rodrigues PC, Beyer U, Schumacher P, Roth T, Fiebig HH (1999). Acid-sensitive polyethylene glycol conjugates of doxorubicin: preparation, *in vitro* efficacy and intracellular distribution. BioorgMed Chem.

[84] Kratz F (2008). Albumin as a drug carrier: design of prodrugs, drug conjugates and nanoparticles. J Control Release.

[85] Coyne CP, Jones T, Sygula A, Bailey J, Pinchuk L (2011). Epirubicin-[anti-HER2/*neu*] synthesized with an epirubicin-(C_13_-imino)-EMCS analog: Anti-neoplastic activity against chemotherapeutic-resistant SKBr-3 mammary carcinoma in combination with organic selenium. Journal of Cancer Therapy.

[86] Inoh K, Muramatsu H, Torii S, Ikematsu S, Oda M (2006). Doxorubicin-conjugated anti-midkine monoclonal antibody as a potential anti-tumor drug. Jpn J Clin Oncol.

[87] Griffiths GL, Mattes MJ, Stein R, Govindan SV, Horak ID (2003). Cure of SCID mice bearing human B-lymphoma xenografts by an anti-CD74 antibody-anthracycline drug conjugate. Clin Cancer Res.

[88] Sapra P, Stein R, Pickett J, Qu Z, Govindan SV (2005). Anti-CD74 antibody-doxorubicin conjugate, IMMU-110, in a human multiple myeloma xenograft and in monkeys. Clin Cancer Res.

[89] Yang HM, Reisfeld RA (1988). Doxorubicin conjugated with a monoclonal antibody directed to a human melanoma-associated proteoglycan suppresses the growth of established tumor xenografts in nude mice. Proc Natl Acad Sci.

[90] Trail PA, Willner D, Lasch SJ, Henderson AJ, Hofstead S (1993). Cure of xenografted human carcinomas by BR96-doxorubicin immunoconjugates. Science.

[91] Diener E, Diner UE, Sinha A, Xie S, Vergidis R (1986). Specific immunosuppression by immunotoxins containing daunomycin. Science.

[92] Dillman RO, Johnson DE, Ogden J, Beidler D (1989). Significance of antigen, drug, and tumor cell targets in the preclinical evaluation of doxorubicin, daunorubicin, methotrexate, and mitomycin-C monoclonal antibody immunoconjugates. Mol Biotherapy.

[93] Page M, Tibeault D, Noel C, Dumas L (1990). Coupling a preactivated daunorubicin derivative to antibody. A new approach. Anti-cancer Research.

[94] Reményi J, Balázs B, Tóth S, Falus A, Tóth G (2003). Isomer-dependent daunomycin release and *in vitro* antitumour effect of cis-aconityl-daunomycin. Biochem Biophys Res Commun.

[95] Ogden JR, Leung K, Kunda SA, Telander MW, Avner BP (1989). Immunoconjugates of doxorubicin and murine antihuman breast carcinoma monoclonal antibodies prepared via an N-hydroxysuccinimide active ester intermediate of cis-aconityl-doxorubicin: preparation and *in vitro* cytotoxicity. Molecular Biotherapy.

[96] Sivam GP, Martin PJ, Reisfeld RA, Mueller BM (1995). Therapeutic efficacy of a doxorubicin immunoconjugate in a preclinical model of spontaneous metastatic human melanoma. Cancer Res.

[97] Coyne CP, Jones T, Pharr T (2011). Synthesis of a covalent gemcitabine-(carbamate)-[anti-HER2/*neu*] immunochemotherapeutic and its cytotoxic anti-neoplastic activity against chemotherapeutic-resistant SKBr-3 mammary carcinoma. Bioorganic and Medicinal Chemistry.

[98] Coyne CP, Jones T, Bear R (2012). Synthesis of Gemcitabine-(C4-*amide*)-[anti-HER2/*neu*] Utilizing a UV-Photoactivated Gemcitabine Intermediate: Cytotoxic Anti-Neoplastic Activity against Chemotherapeutic-Resistant Mammary Adenocarcinoma SKBr-3. Journal of Cancer Therapy: Breast Cancer Special Issue.

[99] Coyne CP, Jones T, Bear R (2013). Gemcitabine-(C4-*amide*)- [anti-HER2/*neu*] Anti- Neoplastic Cytotoxicity in Dual Combination with Mebendazole against Chemotherapeutic-Resistant Mammary Adenocarcinoma. Journal of Clinical and Experimental Oncology.

[100] Kirstein MN, Hassan I, Guire DE, Weller DR, Dagit JW (2006). High-performance liquid chromatographic method for the determination of gemcitabine and 2’,2’-difluorodeoxyuridine in plasma and tissue culture media. J Chromatogr B: Biomed Sci Appl.

[101] Reichelova V, Albertioni F, Liliemark J (1996). Determination of 2-chloro-2’-deoxyadenosine nucleotides in leukemic cells by ion-pair high-performance liquid chromatography. J Chromatogr B: Biomed Sci Appl.

[102] Coyne CP, Jones T, Bear R (2012). Synthesis of a covalent epirubicin-(C(3)-*amide*)-anti-HER2/*neu* immunochemotherapeutic utilizing a UV-photoactivated anthracycline intermediate. Cancer Biotherapy and Radiopharmaceuticals.

[103] Beyer U, Rothen-Rutishauser B, Unger C, Wunderli-Allenspach H, Kratz F (2001). Differences in the intracellular distribution of acid-sensitive doxorubicin-protein conjugates in comparison to free and liposomal formulated doxorubicin as shown by confocal microscopy. Pharmacology Research.

[104] Sinkule JA, Rosen ST, Radosevich JA (1991). Monoclonal antibody 44-3A6 doxorubicin immunoconjugates: comparative *in vitro* anti-tumor efficacy of different conjugation methods. Tumour Biol.

[105] Johnson DA, Briggs SL, Gutowski MC, Barton R (1995). Anti-tumor activity of CC49-doxorubicin immunoconjugates. Anticancer Res.

[106] Stan AC, Radu DL, Casares S, Bona CA, Brumeanu TD (1999). Antineoplastic efficacy of doxorubicin enzymatically assembled on galactose residues of a monoclonal antibody specific for the carcinoembryonic antigen. Cancer Res.

[107] Herbert C, Norris K, Sauk JJ (2003). Targeting of human squamous carcinomas by SPA470-doxorubicin immunoconjugates. J Drug Target.

[108] Zhang Y, Wang N, Li N, Liu T, Dong Z (1992). The antitumor effect of adriamycin conjugated with monoclonal antibody against gastric cancer *in vitro* and *in vivo*. Acta Pharmaceutica Sinica.

[109] Dillman RO, Shawler DL, Johnson DE, Meyer DL, Koziol JA (1986). Preclinical trials with combinations and conjugates of T101 monoclonal antibody and doxorubicin. Cancer Res.

[110] Sjögren HO, Isaksson M, Willner D, Hellström I, Hellström KE (1997). Antitumor activity of carcinoma-reactive BR96-doxorubicin conjugate against human carcinomas in athymic mice and rats and syngeneic rat carcinomas in immunocompetent rats. Cancer Res.

[111] Muldoon LL, Neuwelt EA (2003). BR96-DOX immunoconjugate targeting of chemotherapy in brain tumor models. J Neurooncol.

[112] Muldoon LL (2003). effect of antigenic heterogeneity on the efficacy of enhanced delivery of antibody-targeted chemotherapy in a human lung cancer intracerebral xenograft model in rats. Neurosurgery.

[113] Remsen LG, Trail PA, Hellstrom I, Hellstrom KE, Neuwelt EA (2000). Enhanced delivery improves the efficacy of a tumor-specific doxorubicin immunoconjugate in a human brain tumor xenograft model. Neurosurgery.

[114] Guo P, Ma J, Li S, Guo Z, Adams AL (2001). Targeted delivery of a peripheral benzodiazepine receptor ligand-gemcitabine conjugate to brain tumors in a xenograft model. Cancer Chemother Pharmacol.

[115] Lagisetty P, Vilekar P, Awasthi V (2009). Synthesis of radiolabeled cytarabine conjugates. Bioorganic Med Chem Lett.

[116] Castelli F, Sarpietro MG, Ceruti M, Rocco F, Cattel L (2006). Characterization of lipophilic gemcitabine prodrug-liposomal membrane interaction by differential scanning calorimetry. Mol Pharm.

[117] Guo Z, Gallo JM (1999). Selective Protection of 2’,2’-Difluorodeoxycytidine (Gemcitabine). J Org Chem.

[118] Lau A, Berube G, Ford CH, Gallant M (1995). Novel doxorubicin-monoclonal anti-carcinoembryonic antigen antibody immunoconjugate activity *in-vivo*. Bioorganic and Medicinal Chemistry.

[119] Ali SM, Khan AR, Ahmad MU, Chen P, Sheikh S (2005). Synthesis and biological evaluation of gemcitabine-lipid conjugate (NEO6002). Bioorg Med Chem Lett.

[120] Chen P, Chien PY, Khan AR, Sheikh S, Ali SM (2006). *In-vitro* and *in-vivo* anti-cancer activity of a novel gemcitabine-cardiolipin conjugate. Anticancer Drugs.

[121] Alexander RL, Greene BT, Torti SV, Kucera GL (2005). A novel phospholipid gemcitabine conjugate is able to bypass three drug-resistance mechanisms. Cancer Chemother Pharmacol.

[122] Kiew LV, Cheong SK, Sidik K, Chung LY (2010). Improved plasma stability and sustained release profile of gemcitabine via polypeptide conjugation. Int J Pharm.

[123] Alexander RL, Morris-Natschke SL, Ishaq KS, Fleming RA, Kucera GL (2003). Synthesis and cytotoxic activity of two novel 1-dodecylthio-2-decyloxypropyl-3-phosphatidic acid conjugates with gemcitabine and cytosine arabinoside. J Med Chem.

[124] Alexander RL, Kucera GL (2005). Lipid nucleoside conjugates for the treatment of cancer. Curr Pharm Des.

[125] Shih LB, Goldenberg DM, Xuan H, Lu HW, Mattes MJ (1994). Internalization of an intact doxorubicin immunoconjugate. Cancer Immunol Immunother.

[126] Hansen HJ, Ong GL, Diril H (1996). Internalization and catabolism of radiolabelled antibodies to the MHC class-II invariant chain by B-cell lymphomas. Biochem J.

[127] Pimm MV, Paul MA, Ogumuyiwa Y, Baldwin RW (1988). Biodistribution and tumour localisation of a daunomycin-monoclonal antibody conjugate in nude mice with human tumour xenografts. Cancer Immunol Immunother.

[128] Zhang J, Antonyak MA, Singh G, Cerione RA (2013). A novel mechanism for the up-regulation of EGF-receptor levels in glioblastomas. Cell Rep.

[129] Giordano C, Vizza D, Panza S, Barone I, Bonofiglio D (2013). Leptin increases HER2 protein levels through a STAT3-mediated up-regulation of Hsp90 in breast cancer cells. Mol Oncol.

[130] Wang F, Jiang X, Yang DC, Elliott RL, Head JF (2000). Doxorubicin-gallium-transferrin conjugate overcomes multidrug resistance: evidence for drug accumulation in the nucleus of drug resistant MCF-7/ADR cells. Anticancer Res.

[131] Régina A, Demeule M, Ché C, Lavallée I, Poirier J (2008). Antitumour activity of ANG1005, a conjugate between paclitaxel and the new brain delivery vector Angiopep-2. Br J Pharmacol.

[132] Asakura T, Takahashi N, Takada K, Inoue T, Ohkawa K (1997). Drug conjugate of doxorubicin with glutathione is a potent reverser of multidrug resistance in rat hepatoma cells. Anticancer Drugs.

[133] Mazel M, Clair P, Rousselle C, Vidal P, Scherrmann JM (2001). Doxorubicin-peptide conjugates overcome multidrug resistance. Anticancer Drugs.

[134] Lam W, Leung CH, Chan HL, Fong WF (2000). Toxicity and DNA binding of dextran-doxorubicin conjugates in multidrug-resistant KB-V1 cells: optimization of dextran size. Anticancer Drugs.

[135] Dubikovskaya EA, Torne SH, Pillow TH, Contag CH, Wender PA (2008). Overcoming multidrug resistance of small-molecule therapeutics through conjugation with releasable octaarginine transporters. Proc Natl Acad Sci.

[136] Liu JH, Cao L, Luo PG, Yang ST, Lu F (2010). Fullerene-conjugated doxorubicin in cells. ACS Appl Mater Interfaces.

[137] Widakowich C, Dinh P, de Azambuja E, Awada A, Piccart-Gebhart M (2008). HER-2 positive breast cancer: what else beyond trastuzumab-based therapy?. Anticancer Agents Med Chem.

[138] Medina PJ, Goodin S (2008). Lapatinib: a dual inhibitor of human epidermal growth factor receptor tyrosine kinases. ClinTher.

[139] Cameron DA, Stein S (2008). Drug Insight: intracellular inhibitors of HER2--clinical development of lapatinib in breast cancer. Nat Clin Pract Oncol.

[140] Slamon DJ, Clark GM, Wong SG (1987). Human breast cancer: correlation of relapse and survival with amplification of the HER-2/*neu* oncogene. Science.

[141] Loew S, Schmidt U, Unterberg A, Halatsch ME (2009). The epidermal growth factor receptor as a therapeutic target in glioblastoma multiforme and other malignant neoplasms. Anticancer Agents Med Chem.

[142] Gonzalez-Angulo AM, Morales-Vasquez F, Hortobagyi GN (2007). Overview of resistance to systemic therapy in patients with breast cancer. Adv Exp Med Biol.

[143] Shen H, Lee FY, Gan J (2011). Ixabepilone, a novel microtubule-targeting agent for breast cancer, is a substrate for P-glycoprotein (Pgp/MDR1/ABCB1) but not breast cancer resistance protein (BCRP/ABCG2). J Pharmacol Exp Ther.

[144] Liu F, Liu S, He S, Xie Z, Zu X (2010). Survivin transcription is associated with P-glycoprotein/MDR1 overexpression in the multidrug resistance of MCF-7 breast cancer cells. Oncol Rep.

[145] Patwardhan G, Gupta V, Huang J, Gu X, Liu YY (2010). Direct assessment of P-glycoprotein efflux to determine tumor response to chemotherapy. Biochem Pharmacol.

[146] Pasquier J, Magal P, Boulangé-Lecomte C, Webb G, Foll F (2011). Consequences of cell-to-cell P-glycoprotein transfer on acquired multidrug resistance in breast cancer: a cell population dynamics model. Biol Direct.

[147] Chekhun VF, Zhylchuk VE, Lukyanova NY, Vorontsova AL, Kudryavets YI (2009). Expression of drug resistance proteins in triple-receptor-negative tumors as the basis of individualized therapy of the breast cancer patients. Exp Oncol.

[148] Falchook GS, Duvic M, Hong DS, Wheler J, Naing A, Lim J (2012). Age-stratified phase I trial of a combination of bortezomib, gemcitabine, and liposomal doxorubicin in patients with advanced malignancies. Cancer Chemother Pharmacol.

[149] El Serafi MM, El Khodary AI, El Zawahry HR, Mansour OM, Gaballa HE (2006). Gemcitabine plus doxorubicin as first-line treatment in advanced or metastatic breast cancer (MBC), a phase II study. J Egypt Natl Canc Inst.

[150] Haas NB, Lin X, Manola J, Pins M, Liu G (2012). A phase II trial of doxorubicin and gemcitabine in renal cell carcinoma with sarcomatoid features: ECOG 8802. Med Oncol.

[151] Hensley ML, Wathen JK, Maki RG, Araujo DM, Sutton G (2013). Adjuvant therapy for high-grade, uterus-limited leiomyosarcoma: results of a phase 2 trial (SARC 005). Cancer.

[152] Faivre S, Raymond E, Woynarowski JM, Cvitkovic E (1999). Supraadditive effect of 2’,2’-difluorodeoxycytidine (gemcitabine) in combination with oxaliplatin in human cancer cell lines. Cancer Chemother Pharmacol.

[153] Correale P, Cerretani D, Marsili S, Pozzessere D, Petrioli R (2003). Gemcitabine increases systemic 5-fluorouracil exposure in advanced cancer patients. Eur J Cancer.

[154] Mey V, Giovannetti E, De Braud F, Nannizzi S, Curigliano G (2006). *In vitro* synergistic cytotoxicity of gemcitabine and pemetrexed and pharmacogenetic evaluation of response to gemcitabine in bladder cancer patients. Br J Cancer.

[155] Giovannetti E, Mey V, Danesi R, Mosca I, Del Tacca M (2004). Synergistic cytotoxicity and pharmacogenetics of gemcitabine and pemetrexed combination in pancreatic cancer cell lines. Clin Cancer Res.

[156] Wong SJ, Myette MS, Wereley JP, Chitambar CR (1999). Increased sensitivity of hydroxyurea-resistant leukemic cells to gemcitabine. Clin Cancer Res.

[157] Zhou B, Mi S, Mo X, Shih J, Tsai J (2002). Time and sequence dependence of hydroxyurea in combination with gemcitabine in human KB cells. Anticancer Res.

[158] Kamat AM, Karashima T, Davis DW, Lashinger L, Bar-Eli M (2004). The proteasome inhibitor bortezomib synergizes with gemcitabine to block the growth of human 253JB-V bladder tumors *in vivo*. Mol Cancer Ther.

[159] Ricciardi S, Mey V, Nannizzi S, Pasqualetti G, Crea F (2010). Synergistic cytotoxicity and molecular interaction on drug targets of sorafenib and gemcitabine in human pancreas cancer cells. Chemotherapy.

[160] Coyne CP, Jones T, Bear R (2012). Influence of Alternative Tubulin Inhibitors on the Potency of a Epirubicin-Immunochemotherapeutic Synthesized with an Ultra Violet Light-Activated Intermediate. Journal of Cancer and Clinical Oncology.

[161] Jin R, Moreira Teixeira LS, Krouwels A, Dijkstra PJ, van Blitterswijk CA (2010). Synthesis and characterization of hyaluronic acid-poly(ethylene glycol) hydrogels via Michael addition: An inject-able biomaterial for cartilage repair. Acta Biomater.

[162] Fry AK, Schilke KF, McGuire J, Bird KE (2010). Synthesis and anticoagulant activity of heparin immobilized "end-on" to polystyrene microspheres coated with end-group activated polyethylene oxide. J Biomed Mater Res B Appl Biomater.

[163] Coyne CP, Fenwick BW, Ainsworth J (1997). Cytotoxic activity of doxorubicin "loaded" *neu*trophils against human mammary carcinoma (HTB-19). Biotherapy.

[164] Shen WC, Ryser HJ (1981). cis-Aconityl spacer between daunomycin and macromolecular carriers: a model of pH-sensitive linkage releasing drug from a lysosomotropic conjugate. Biochem Biophys Res Commun.

[165] Aboud-Pirak E, Hurwitz E, Bellot F, Schlessinger J, Sela M (1989). Inhibition of human tumor growth in nude mice by a conjugate of doxorubicin with monoclonal antibodies to epidermal growth factor receptor. Proc Natl Acad Sci.

[166] Michalski CW, Erkan M, Sauliunaite D, Giese T, Stratmann R (2008). *Ex vivo* chemosensitivity testing and gene expression profiling predict response towards adjuvant gemcitabine treatment in pancreatic cancer. Br J Cancer.

[167] Hoang T, Kim K, Jaslowski A, Koch P, Beatty P (2003). Phase II study of second-line gemcitabine in sensitive or refractory small cell lung cancer. Lung Cancer.

[168] Bierau J, van Gennip AH, Leen R, Meinsma R, Caron HN (2006). Cyclopentenyl cytosine-induced activation of deoxycytidine kinase increases gemcitabine anabolism and cytotoxicity in neuroblastoma. Cancer Chemother Pharmacol.

[169] Santini V, D’Ippolito G, Bernabei PA, Zoccolante A, Ermini A (1996). effects of fludarabine and gemcitabine on human acute myeloid leukemia cell line HL 60: direct comparison of cytotoxicity and cellular Ara-C uptake enhancement. Leuk Res.

[170] Yang HM, Reisfeld RA Pharmacokinetics and mechanism of action of a doxorubicin-monoclonal antibody 9.2.27 conjugate directed to a human melanoma proteoglycan. J Natl Cancer Inst.

[171] Lutsenko SV, Feldman NB, Severin SE (2002). Cytotoxic and antitumor activities of doxorubicin conjugates with the epidermal growth factor and its receptor-binding fragment. J Drug Target.

[172] Mueller H, Kassack MU, Wiese M (2004). Comparison of the usefulness of the MTT, ATP, and calcein assays to predict the potency of cytotoxic agents in various human cancer cell lines. Biomolecular Screening.

[173] Ulukaya E, Ozdikicioglu F, Oral AY, Dermirci M (2008). The MTT assay yields a relatively lower result of growth inhibition than the ATP assay depending on the chemotherapeutic drugs tested. Toxicol *In Vitro*.

[174] Varache-Lembège M, Larrouture S, Montaudon D, Robert J, Nuhrich A (2008). Synthesis and antiproliferative activity of aryl- and heteroaryl-hydrazones derived from xanthone carbaldehydes. Eur J Med Chem.

[175] Kars MD, Iseri OD, Gunduz U, Molnar J (2008). Reversal of multidrug resistance by synthetic and natural compounds in drug-resistant MCF-7 cell lines. Chemotherapy.

[176] Huang H, Pierstorf E, Osawa E, Ho D (2007). Active nanodiamond hydrogels for chemotherapeutic delivery. Nano Lett.

[177] Dery MC, Van Themsche C, Provencher D, Mes-Masson AM, Asselin E (2007). Characterization of EN-1078D, a poorly differentiated human endometrial carcinoma cell line: a novel tool to study endometrial invasion *in vitro*. Reprod Biol Endocrinol.

[178] Spee B, Jonkers MD, Arends B, Rutteman GR, Rothuizen J (2006). Specific down-regulation of XIAP with RNA interference enhances the sensitivity of canine tumor cell-lines to TRAIL and doxorubicin. Mol Cancer.

[179] Denora N, Laquintana V, Trapani A, Lopedota A, Latrofa A (2010). Translocator protein (TSPO) ligand-Ara-C (cytarabine) conjugates as a strategy to deliver antineoplastic drugs and to enhance drug clinical potential. Mol Pharm.

[180] Martarelli D, Pompei P, Baldi C, Mazzoni G (2008). Mebendazole inhibits growth of human adrenocortical carcinoma cell lines implanted in nude mice. Cancer Chemother Pharmacol.

[181] Pourgholami MH, Akhter J, Wang L, Lu Y, Morris DL (2005). Antitumor activity of albendazole against the human colorectal cancer cell line HT-29: *in vitro* and in a xenograft model of peritoneal carcinomatosis. Cancer Chemother Pharmacol.

[182] Morris DL, Jourdan JL, Pourgholami MH (2001). Pilot study of albendazole in patients with advanced malignancy. effect on serum tumor markers/high incidence of *neu*tropenia. Oncology.

[183] Pourgholami MH, Woon L, Almajd R, Akhter J, Bowery P (2001). *In vitro* and *in vivo* suppression of growth of hepatocellular carcinoma cells by albendazole. Cancer Lett.

[184] Khalilzadeh A, Wangoo KT, Morris DL, Pourgholami MH (2007). Epothilone-paclitaxel resistant leukemic cells CEM/dEpoB300 are sensitive to albendazole: Involvement of apoptotic pathways. Biochem Pharmacol.

[185] Spagnuolo PA, Hu J, Hurren R, Wang X, Gronda M (2010). The antihelmintic flubendazole inhibits microtubule function through a mechanism distinct from Vinca alkaloids and displays pre-clinical activity in leukemia and myeloma. Blood.

[186] Mukhopadhyay T, Sasaki J, Ramesh R, Roth JA (2002). Mebendazole elicits a potent antitumor effect on human cancer cell lines both *in vitro* and *in vivo*. Clin Cancer Res.

[187] Sasaki J, Ramesh R, Chada S, Gomyo Y, Roth JA (2002). The anthelmintic drug mebendazole induces mitotic arrest and apoptosis by depolymerizing tubulin in non-small cell lung cancer cells. Mol Cancer Ther.

[188] Doudican N, Rodriguez A, Osman I, Orlow SJ (2008). Mebendazole induces apoptosis via Bcl-2 inactivation in chemoresistant melanoma cells. Mol Cancer Res.

[189] Pourgholami MH, Yan Cai Z, Lu Y, Wang L, Morris DL (2011). Albendazole: a potent inhibitor of vascular endothelial growth factor and malignant ascites formation in OVCAR-3 tumor-bearing nude mice. Clin Cancer Res.

[190] Pourgholami MH, Cai ZY, Badar S, Wangoo K, Poruchynsky MS (2010). Potent inhibition of tumoral hypoxia-inducible factor 1alpha by albendazole. BMC Cancer.

[191] Pourgholami MH, Cai ZY, Wang L, Badar S, Links M (2009). Inhibition of cell proliferation, vascular endothelial growth factor and tumor growth by albendazole. Cancer Invest.

[192] Nianjun H, Cerepnalkoski L, Nwankwo JO, Dews M, Landolph JR (1994). Induction of chromosomal aberrations, cytotoxicity, and morphological transformation in mammalian cells by the antiparasitic drug flubendazole and the antineoplastic drug harringtonine. Fundam Appl Toxicol.

